# Integrated Optimization, Genomic Characterization, and Functional Evaluation of Biogenic Selenium Nanoparticles from *Bacillus licheniformis* BLN313: Antibacterial and Anticancer Potential

**DOI:** 10.3390/ijms27177792

**Published:** 2026-08-31

**Authors:** Shiza Nawaz, Maryam Anayat, Sana Parveen, Nida Nawaz, Alhassan Alrafaie, Yingjie Wang, Fenghuan Wang

**Affiliations:** 1 Key Laboratory of Geriatric Nutrition and Health, Beijing Technology and Business University, Ministry of Education, Beijing 100048, China; shizanawaz962@gmail.com (S.N.); maryamanayat135@gmail.com (M.A.); 1970201307@st.btbu.edu.cn (N.N.); 15931338273@163.com (Y.W.); 2 Key Laboratory of Digital-Intelligence and Dynamic Perception for Food Quality of China Light Industry, Beijing Technology and Business University, Beijing 100048, China; 3 Industrial Biotechnology Division, National Institute for Biotechnology and Genetic Engineering College, Pakistan Institute of Engineering and Applied Sciences (NIBGE-C, PIEAS), Jhang Road, Faisalabad 38000, Pakistan; sana382p@gmail.com; 4 Department of Medical Laboratory, College of Applied Medical Sciences in Al-Kharj, Prince Sattam Bin Abdulaziz University, Al-Kharj 11942, Saudi Arabia; a.alrafaie@psau.edu.sa; 5 Hubei Key Laboratory of Selenium Resource Research and Biological Application, Hubei Minzu University, Enshi 445000, China

**Keywords:** *Bacillus licheniformis*, SeNPs, ANI/dDDH, phylogenomics, comparative genomics, selenium-associated genes

## Abstract

Microbial synthesis of selenium nanoparticles (SeNPs) offers a sustainable alternative to chemical routes, but the genetic basis of selenium handling in *Bacillus* remains poorly defined, which limits rational strain selection. Here, SeNP production, physicochemical characterization, and closed-genome sequencing are combined for *Bacillus licheniformis* BLN313. Selenite reduction peaked at 500 µg/mL Na_2_SeO_3_ (88.8% conversion; 444 ± 27 µg/mL Se^0^); at higher concentrations, conversion efficiency and viability diverged, indicating that tolerance and reductive capacity are distinct traits. Purified SeNPs were spherical and partially crystalline trigonal Se^0^ (TEM 190 ± 52 nm; DLS 166 nm, PDI 0.03; zeta potential −20.8 mV), carrying a proteinaceous capping layer confirmed by XPS, EDS, and FTIR and shown by LC-MS to be enriched in cell wall-derived metabolites. The particles were bactericidal against *Micrococcus luteus* (MIC 62.5 µg/mL) and *Klebsiella pneumoniae* (MIC 250 µg/mL) and reduced MCF-7 viability (IC50 2.7 µg/mL) while sparing MCF-10A cells. The 4.11 Mb genome (46.3% GC; ANI 99.7%, dDDH 97.8%) encodes SulP and Pit transporters, multiple *trxB* copies, and sulfur-metabolism and oxidative-stress genes, defining a candidate gene set for selenium uptake, reduction and detoxification. BLN313 thus provides a genetically defined platform for SeNP production in biomedical and environmental applications.

## 1. Introduction

Selenium (Se) is an essential micronutrient that possess remarkably superior biological activity upon alteration into nanoscale elemental selenium nanoparticles (SeNPs). These SeNPs have a broad range of applications, from serving as potent antioxidants and effective antimicrobial agents to therapeutics candidates in the health sector. Recent studies underscore the substantial antibacterial efficacy of these agents against clinically significant bacterial pathogens, highlighting their pronounced anti-biofilm characteristics, which augment their potential as nano-enabled antimicrobial agents [1,2]. Concurrently, SeNPs have demonstrated promising antiproliferative effects on various cancer cell lines, further emphasizing their increasing significance as versatile therapeutic nanomaterials [3,4].

In addition to biomedical applications, SeNPs have expanding roles as efficacious biocontrol agents and plant growth regulators in agriculture. Moreover, they also serve as sustainable catalysts in environmental remediation [5,6,7]. Biogenic nanoparticles (NPs) exhibit enhanced biocompatibility and less toxicity compared to their physical and chemical counterparts owing to the inherent biomolecular capping. Furthermore, the SeNPs’ bioactivity is strictly governed by the nanoparticle size, morphology, and synthesis origin, thus emphasizing the significance of optimized biosynthesis protocols for enhanced bioactivity [7,8,9].

Microbial-mediated synthesis of SeNPs is particularly well documented in *Bacillus* species, exhibiting inherent reductive ability to aerobically transform toxic selenite form (Se^4+^) to elemental selenium form (Se^0^) [9,10]. Process optimization has revealed that initial Se salt concentration critically modulates the microbial growth kinetics, NP yield, and resultant particle size, with near complete selenite bioconversion attained at millimolar concentrations [9]. Nevertheless, the crucial underlying molecular pathways regulating selenite reduction, nanoparticle capping and stabilization, and cellular stress adaptation have yet to be fully established in *Bacillus* species [11].

Despite growing interest in microbial SeNP biosynthesis, most previous studies have primarily focused on physiological responses, transcriptomic regulation, or process optimization, while genome-level insights into selenium-metabolizing *Bacillus* strains remain limited. In particular, the genetic basis underlying selenium oxyanion transport, intracellular reduction, and detoxification in *Bacillus licheniformis* remains poorly understood [9,11]. In this context, the present study provides the first genome-wide characterization of *B. licheniformis* strain BLN313 with experimentally confirmed SeNP-producing capability. Comparative genomic analysis was used to identify and integrate candidate genes involved in selenium uptake (e.g., SulP and Pit transporters), redox metabolism (thioredoxin system and sulfur-associated enzymes), and oxidative stress response, thereby establishing a genomic framework linking genotype to the SeNP-producing phenotype in this strain.

In addition to the genomic investigation, this study aimed to optimize SeNP biosynthesis in strain BLN313 and comprehensively characterize the physicochemical properties of the synthesized nanoparticles using advanced analytical techniques. The biological potential of the SeNPs was further evaluated through antibacterial assays against *Micrococcus luteus* (*M. luteus*) and *Klebsiella pneumoniae* (*K. pneumoniae*), as well as anticancer activity against MCF-7 breast cancer cells. Collectively, these integrated approaches provide mechanistic and application-oriented insights into SeNP biosynthesis in BLN313 and support the development of sustainable and scalable microbial platforms for selenium nanoparticle production, with potential biomedical applications.

## 2. Results

### 2.1. Growth Dynamics of Strain BLN313 Under Sodium Selenite Stress

SeNP biosynthesis is greatly influenced by the concentration of Se substrate and has been extensively reported in diverse bacterial species including *Bacillus*, *Staphylococcus*, *Pseudomonas* and *lactic acid* bacteria, exhibiting dose-dependent selenite reduction and SeNP biosynthesis [7,8,10,12]. To identify the optimal sodium selenite concentration for achieving maximal SeNP production and to ensure bacterial viability during biotransformation processes, the strain BLN313 was cultivated in different concentrations of sodium selenite ranging from 100 µg/mL to 10 mg/mL. Before conducting the experiments, the strain was activated, which showed a characteristic *Bacillus*-like colony morphology on LB agar plates (Supplementary Figure S1).

**Figure 1:**
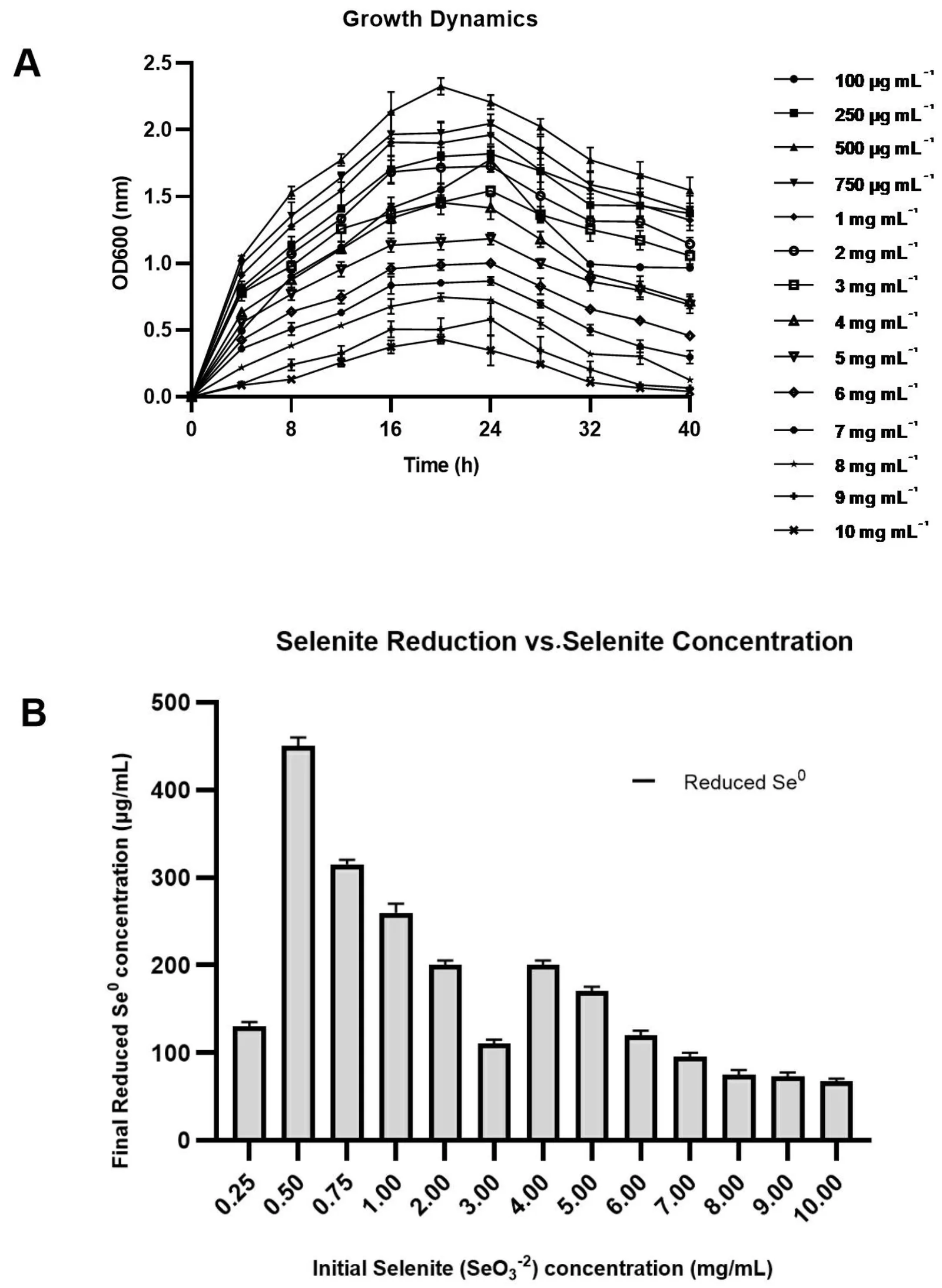
Growth dynamics of strain BLN313 and quantification of reduced Se^0^ at different concentrations of Na_2_SeO_3_ stress. (**A**) Effect of different concentrations of selenite stress (100–10 mg/mL) on BLN313 cell growth cultivated for 40 h at 37 °C. Data represent the mean ± SD (*n* = 3). (**B**) Relationship between initial selenite concentration (0.25–10 mg/mL) and ultimate reduced Se^0^ concentration. Data represent the mean ± SD (*n* = 3).

Figure 1A demonstrates the growth kinetics of strain BLN313 at varying sodium selenite concentrations. At lower-dose conditions (100–750 µg/mL), the strain exhibited a short lag phase over a range of 0 to 4 h, preceded by a pronounced exponential growth-period ranging from 4 to 16 h, and later entered the stationary-state after 16 h. Of these, the 500 µg/mL concentration supported the maximum biomass production, with an OD_600_ of nearly 2.3 at 24 h. A further increase in sodium selenite concentrations (1–5 mg/mL) resulted in growth inhibition in a dose-dependent manner, characterized by extended lag phases and decreased biomass production. However, active growth was still observable up to 5 mg/mL, with an absorbance value of 1.2 units (OD_600_) at 20 h, whereas the growth was greatly inhibited at a higher concentration interval ranging from 6 mg/mL to 10 mg/mL), showing prolonged lag phases and reduced exponential growth. At 10 mg/mL, growth inhibition was maximally suppressed, exhibiting OD_600_ values near baseline throughout the incubation period. Overall, BLN313 exhibited relatively high tolerance to sodium selenite stress, with robust growth maintained between 500 and 750 µg/mL range, highlighting the potential of BLN313 for selenium biotransformation under elevated selenite concentrations. OD_600_ readings were blank-corrected using uninoculated selenite-containing medium. However, the values may still include light scattering from SeNPs formed in situ and should therefore be interpreted as a measure of overall culture turbidity rather than a direct estimate of biomass.

### 2.2. Selenite Reduction and Se^0^ Accumulation Profile in Strain BLN313

The reduction ability of strain BLN313 was greatly influenced by varying selenite substrate concentrations, showing a steep optimum range for Se^0^ biosynthesis, as shown in Figure 1B. At an initial sodium selenite concentration of 0.5 mg/mL, the highest selenite reduction was observed, attaining a maximum reduction efficiency of 88.8% and a mean Se^0^ production amount of 444 ± 27 µg/mL. However, any further increase in selenite concentrations led to a clear dose-dependent decline in reduction efficiency, indicating substrate inhibition. The reduction efficiency declined to 25.00% at a 1.0 mg/mL concentration of selenite, while at a 5.0 mg/mL concentration, it decreased sharply to only 3.02%, corresponding to 151.03 ± 4.45 µg/mL Se^0^ concentration. At 8.0 mg/mL, Se^0^ production was nearly suppressed, with a reduction efficiency of 0.193% only. Overall, these results show that strain BLN313 displays efficient Se^0^ synthesis at selenite concentrations up to 5.0 mg/mL, whereas higher concentrations greatly reduce the reduction efficiency, representing the toxic effect of substrate overload on bacterial growth.

### 2.3. Biogenic SeNP Purification and Physiochemical Characterization

A detailed characterization of biogenic selenium nanoparticles (SeNPs) synthesized by strain BLN313 at a sodium selenite concentration of 500 µg/mL was carried out by employing a combination of analytical techniques and microscopic-based analysis. These nanoparticles were synthesized intracellularly through toxic selenite ion (Se^4+^) reduction to non-toxic elemental SeNPs (Se^0^). Then, these synthesized SeNPs were recovered and purified following the protocol with minor modifications introduced to improve the bacterial cell lysis efficiency [13]. Supplementary Figure S2 exhibits the characteristic reddish pigmentation in the aqueous phase, indicating the successful isolation of SeNPs.

**Figure 2:**
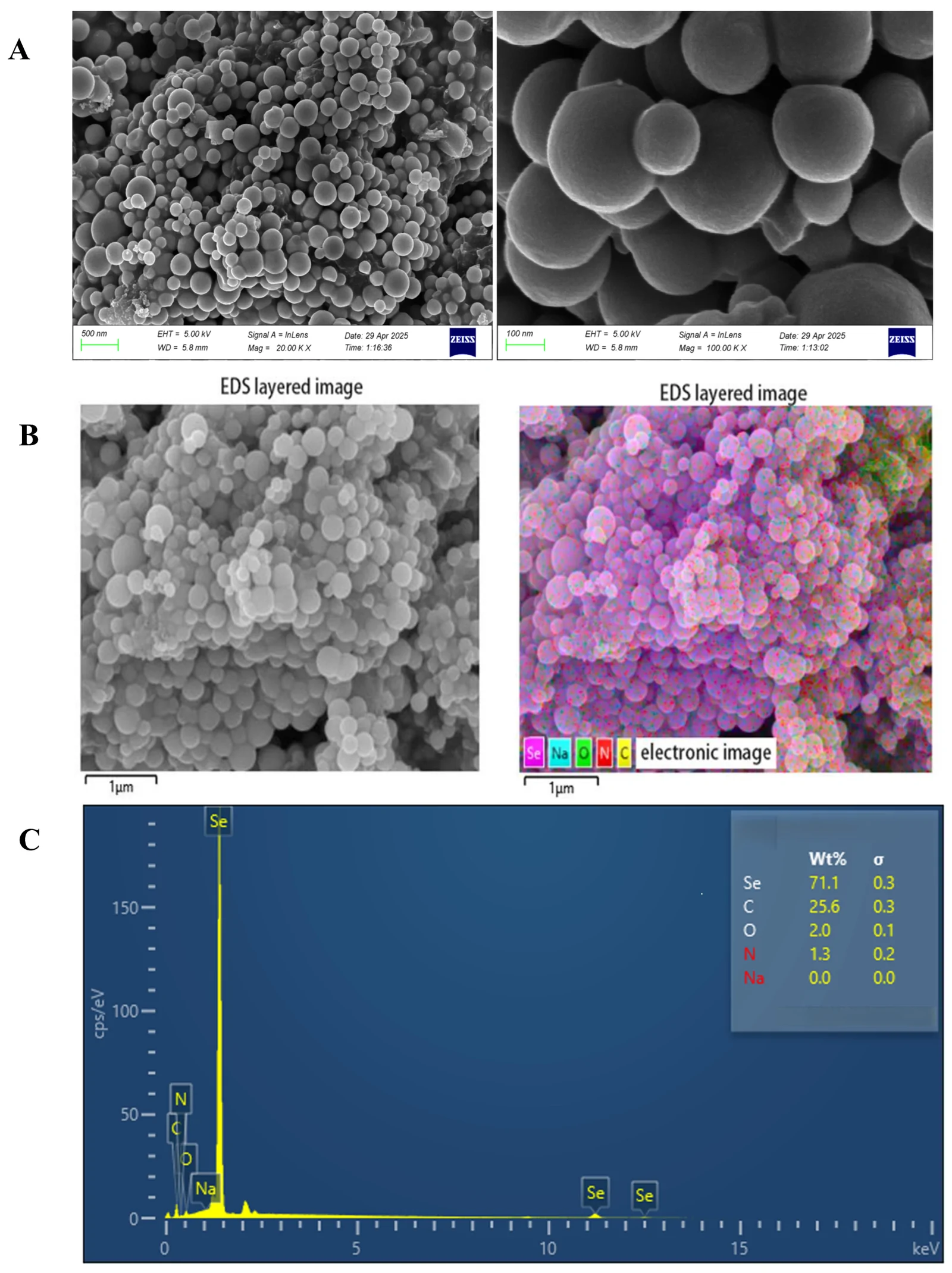
Scanning electron microscopy (SEM) and energy-dispersive X-ray spectroscopy (EDS) characterization of selenium nanoparticles (SeNPs) synthesized by strain BLN313 at a selenite concentration of 500 µg/mL. (**A**) SEM micrographs at various magnifications revealing the predominantly spherical morphology of the SeNPs. (**B**,**C**) EDS layered image and corresponding spectrum confirming the presence and spatial distribution of Se atoms, along with associated elements including Na, C, N, and O on the SeNPs.

To investigate the morphological and particle size characteristics of SeNPs synthesized by strain BLN313, electron microscopy analysis was employed. Scanning electron microscopy (SEM) images indicated the synthesis of predominantly spherical SeNPs at the nanoscale, having a relatively smooth surface and moderate aggregation (Figure 2A). Energy-dispersive X-ray spectroscopy (EDS) spectra further validated selenium as the principal elemental component (71.1 wt %) in the nanoparticles, thereby confirming successful biogenic formation. In addition, the minor signals for C, O, and N presence were also noticed, possibly corresponding to biomolecular capping layers associated with the surface of nanoparticles (Figure 2B,C).

**Figure 3:**
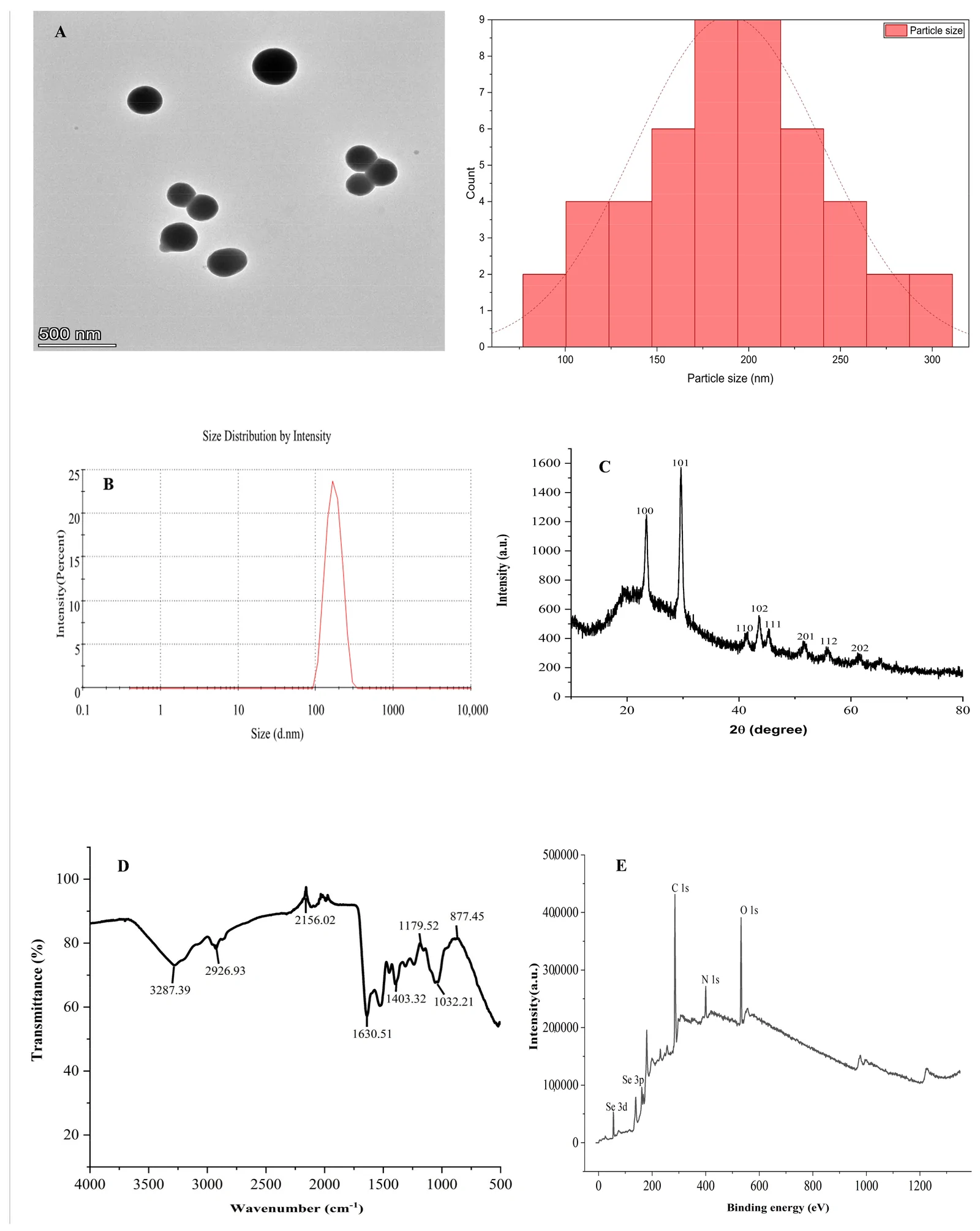
Characterization of biogenic selenium nanoparticles (SeNPs) produced by BLN313 at a selenite concentration of 500 µg/mL. (**A**) Transmission electron microscopy (TEM) micrographs showing the spherical morphology and nanoscale size distribution of the SeNPs. (**B**) Dynamic light scattering (DLS) intensity profile illustrating the hydrodynamic size distribution of the SeNPs in suspension. (**C**) X-ray diffraction (XRD) pattern confirming the crystalline trigonal structure of the nanoparticles. (**D**) Fourier-transform infrared (FTIR) spectra indicating the characteristic surface functional groups associated with SeNPs, including hydroxyl, amide, and polysaccharide-related vibrations. (**E**) X-ray photoelectron spectroscopy (XPS) survey spectrum displaying the elemental composition of the SeNP surface, including Se 3d, C 1s, N 1s, and O 1s signals.

Transmission electron microscopy (TEM) observations further verified that these biosynthesized SeNPs were primarily spherical, distinct, and clearly bounded while exhibiting moderate aggregation, which is a typical feature of biogenic SeNPs. Particle size analysis exhibited a polydisperse distribution of SeNPs with an average diameter of 190 ± 52 nm (Figure 3A). Dynamic light scattering (DLS) and zeta potential measurements were used to assess the hydrodynamic size and surface charge of BLN313-SeNPs. The DLS intensity profile revealed a single predominant population (Figure 3B), and triplicate measurements yielded a mean Z-average diameter of 165.8 ± 0.4 nm, indicating monodisperse and well-dispersed particle distribution in suspension. The mean zeta potential was −20.8 ± 0.2 mV, reflecting a moderately negative surface charge consistent with values reported for stable biogenic SeNP suspensions [14]. Full triplicate DLS and zeta potential data are provided in Supplementary Table S2.

X-Ray Diffraction (XRD) analysis verified the presence of crystalline trigonal Se^0^ in BLN313-SeNPs, as depicted in Figure 3C. The dominant (101) reflection, along with well-resolved higher-angle peaks, substantiates the existence of a crystalline Se^0^ phase. A notable diffuse background beneath the crystalline reflections suggests a significant noncrystalline/amorphous contribution, likely due to organic biomolecular surface residues, consistent with observations in other biogenic SeNP preparations [15,16]. A semi-quantitative assessment, derived from the ratio of integrated Bragg reflection area to total integrated scattered intensity after background correction, indicated an apparent crystallinity degree of approximately 29% implying about 71% diffuse/noncrystalline contribution on an area basis. This represents a relative, area-based estimate rather than an absolute weight fraction. In summary, the XRD data confirm that BLN313-SeNPs predominantly consist of a diffuse/noncrystalline component, alongside crystalline trigonal Se^0^ reflections.

Fourier-Transform Infrared (FTIR) spectroscopy was conducted to investigate the surface functional groups of BLN313-SeNPs and to evaluate the possible contribution of bacterial biomolecules to nanoparticle capping and stabilization. The SeNP spectrum exhibited bands at 3287.39, 2926.93, 1630.51, 1403.32, and 1179.52/1032.21 cm^−1^, corresponding to hydrogen-bonded O–H stretching with possible N–H contribution, aliphatic C–H stretching, amide I/C=O stretching, O–H/C–H bending with possible carboxylate contribution, and C–O/C–N/C–O–C vibrations, respectively (Figure 3D). Similar features have been reported for biogenic SeNP preparations and associated microbial biomolecules [15,17]. To differentiate SeNP-associated signals from background bacterial components, a blank bacterial sample prepared without selenite was analyzed as a control (Supplementary Figure S3). The control spectrum exhibited bands at 3297.67, 2998.51, 1652.30, 1546.63, 1401.99, 1180.22, 1070.29, and 933.37 cm^−1^. The spectral overlap between the two samples suggests the association of bacterial biomolecules with the nanoparticle surface, while minor shifts in the O–H, amide/C=O, and C–O regions may reflect their interaction with the SeNP surface. The FTIR data thus support the involvement of bacterial protein- and polysaccharide-like biomolecules in the capping and stabilization of BLN313-SeNPs.

X-Ray Photoelectron Spectroscopy (XPS) analysis was performed to examine the surface elemental composition of BLN313-SeNPs (Figure 3E). The survey spectrum showed signals corresponding to Se 3d (55.0 eV), C 1s (285.0 eV), N 1s (400.0 eV), and O 1s (532.0 eV). The Se 3d signal near 55.0 eV supports the presence of Se^0^, consistent with reported Se 3d_5_/_2_ binding energies for bacterially synthesized SeNPs [18]. The C 1s, N 1s, and O 1s signals indicate the presence of carbon-, nitrogen-, and oxygen-containing surface species, which are likely associated with bacterial biomolecules on the nanoparticle surface. These findings are consistent with the FTIR results and support the association of protein- and polysaccharide-like biomolecules with BLN313-SeNPs. Overall, the XPS survey confirms the presence of selenium and surface-associated biomolecular components in the synthesized nanoparticles. Deconvoluted high-resolution XPS spectra are provided in Supplementary Figure S4.

### 2.4. Surface Metabolite Profiling of Biogenic SeNPs by UHPLC-ESI-Orbitrap-MS

Untargeted UHPLC-ESI-Q Exactive Orbitrap-MS profiling of purified nanoparticles, compared to an identically processed wash control, revealed metabolites enriched on the nanoparticle surface. Six metabolites were selected for detailed analysis including muramic acid, UDP-N-acetylmuraminate, 5′-methylthioadenosine, L-lysine, pipecolic acid, and L-phenylalanine, all exhibiting higher relative signals on the nanoparticles. Particularly, muramic acid and UDP-N-acetylmuraminate are associated with peptidoglycan structure and biosynthesis, suggesting that the particles are capped by biomolecules from the cell envelope. This finding was accompanied by the presence of amino acid and sulfur-related metabolites (L-lysine, pipecolic acid, 5′-methylthioadenosine, and L-phenylalanine), which is consistent with a proteinaceous corona and corroborated by the amide and polysaccharide bands observed via FTIR. The annotations are putative (MSI Level 2), supported by accurate mass, retention time, isotope pattern, and MS^2^ fragmentation (Supplementary Figure S5).

### 2.5. Antibacterial Activity of SeNPs

SeNPs were active against both test organisms, with the Gram-positive *M. luteus* consistently more susceptible than the Gram-negative *K. pneumoniae*. In disk diffusion, SeNPs produced inhibition zones of 13.23 ± 0.15 mm against *M. luteus* and 9.23 ± 0.15 mm against *K. pneumoniae* (mean ± SD, *n* = 3) versus 31.17 ± 0.09 mm and 30.73 ± 0.18 mm for ciprofloxacin, while the water and selenite controls gave no zone beyond the 6 mm disc. By two-way ANOVA with Tukey’s test, SeNP zones were significantly smaller than ciprofloxacin (*p* < 0.001) but significantly larger against *M. luteus* than *K. pneumoniae* (*p* < 0.01). The same pattern was observed in the broth microdilution: *M. luteus* (MIC 62.5, MBC 125 µg/mL) was more susceptible than *K. pneumoniae* (MIC 250, MBC 500 µg/mL), and an MBC/MIC ratio of 2 for both species indicates bactericidal activity. MIC and MBC were not determined for ciprofloxacin, which served as a disk diffusion positive control only, nor for the negative control. As MIC and MBC are two-fold dilution endpoints, they are reported as values rather than analyzed by ANOVA. All parameters are summarized in Table 1.

### 2.6. Cell Viability and Anticancer Activity of SeNPs

**Figure 4:**
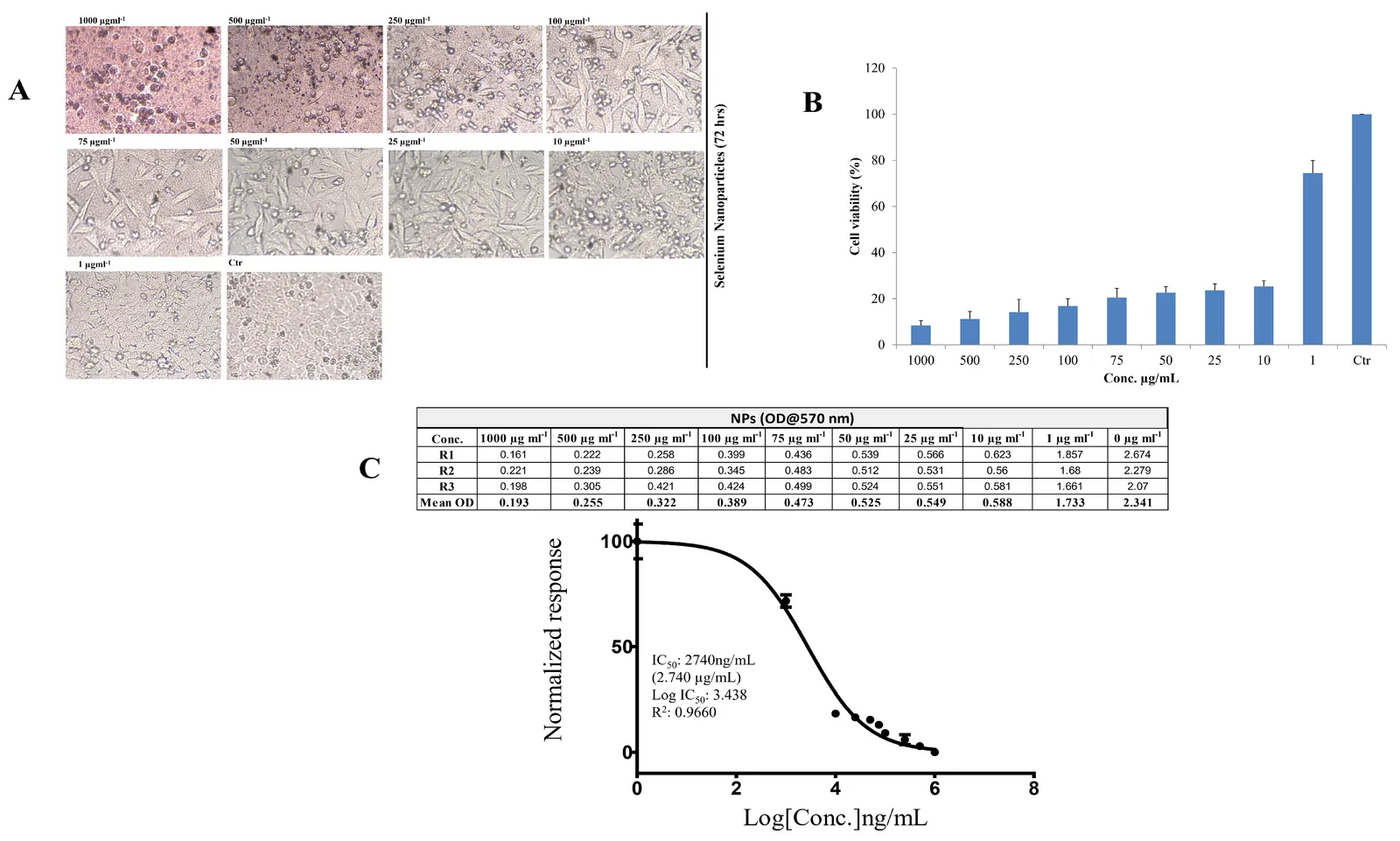
Assessment of the cytotoxic effects of biogenic SeNPs on MCF-7 cells utilizing the MTT assay. MCF-7 cells were treated with SeNPs at concentrations ranging from 1 to 1000 µg/mL over a 72-h period. (**A**) Phase-contrast images obtained using an inverted microscope (IMT-2; Olympus, Tokyo, Japan) at a total magnification of 100× (10× eyepiece × 10× objective) demonstrate reduced cell confluence and increased cell rounding/detachment at higher SeNP concentrations compared to the untreated control. (**B**) Cell viability (%) relative to the untreated control (set at 100%) was determined as 100 × (A570 sample/A570 control). (**C**) Absorbance measurements at 570 nm (A570) for cells exposed to SeNPs at various concentrations, presenting data from three independent experiments (R1–R3; *n* = 3), with each performed in triplicate wells, and a nonlinear dose–response curve was utilized to determine the IC50 value. Data are presented as the mean ± SD from three independent experiments (*n* = 3).

BLN313-SeNPs decreased MCF-7 cell viability in a concentration-dependent manner over 72 h. Phase-contrast microscopy showed gradual morphological changes with increasing nanoparticle concentrations, including cell rounding, detachment, and reduced monolayer confluence compared to untreated controls (Figure 4A). MTT assays confirmed this observation when viability dropped from 74.5 ± 3.2% at 1 µg/mL to 25.3 ± 2.1% at 10 µg/mL and 8.4 ± 1.5% at 1000 µg/mL Figure 4B. All treated groups differed significantly from the control (one-way ANOVA with Dunnett’s post hoc test, *p* < 0.001). The dose–response curve fitted by four-parameter nonlinear regression (Figure 4C) provided an IC_50_ value of 2.74 µg/mL, with a 95% confidence interval of 2.35–3.14 µg/mL and R^2^ = 0.966. In Figure 4C, each data point represents the mean of three independent experiments (each performed in triplicate), and the fitted curve illustrates the nonlinear decline in cell viability with increasing SeNP concentration, allowing for reliable determination of the IC_50_. BLN313-SeNPs maintained MCF-10A normal mammary epithelial cell viability above 97% across the entire concentration range tested (1–1000 µg/mL; Supplementary Figure S6).

### 2.7. Genome Sequencing and Assembly Statistics

Illumina paired-end sequencing yielded 7,541,164 reads (1.14 Gb) with a GC content of 46.41%. The sequencing data demonstrated high quality, as indicated by Q20 and Q30 scores of 98.89% and 96.66%, respectively. After quality filtering, 7,501,648 high-quality reads (99.48%) were preserved, corresponding to 1.13 Gb of clean data and an estimated 275X genome coverage. PacBio sequencing generated a total of 898,158 reads, comprising 8.41 Gb of sequence data. The longest read length was 246,407 bp, with a 10,103 bp N50 read length, and no ambiguous bases were detected. Genome size estimation based on 19-mer frequency analysis predicted a genome size of approximately 4.06 Mb, with a repetitive fraction of 1.02%, indicating low genomic redundancy (Table 2).

Hybrid genome assembly of Illumina short reads and PacBio long reads was conducted by using Unicycler v0.4.8 and Flye v2.9.1, followed by assembly polishing with Pilon v1.18, resulting in a complete single circular chromosome having 4,114,473 bp with 46.28% of GC content. No evidence of plasmids was noticed. Assessment of potential contamination using the ContEst16S tool implemented on the EzBioCloud platform classified the assembly status as undetermined (Table 2).

### 2.8. Non-Coding RNA Prediction

Genome-wide analysis of strain BLN313 identified a complete set of non-coding RNAs, consisting of all essential tRNA genes and the conserved 23S, 16S, and 5S rRNA genes, facilitating efficient protein synthesis and active metabolism in BLN313 (Table 3). Additionally, several regulatory non-coding RNAs were also detected, suggesting their post-transcriptional control that may help adjust redox balance and stress responses, particularly under selenium exposure (Table 3). These findings underline the role of non-coding RNAs in maintaining genome stability and enabling adaptive potential in BLN313.

### 2.9. Genome Annotation and Subsystem Classification of BLN313

**Figure 5:**
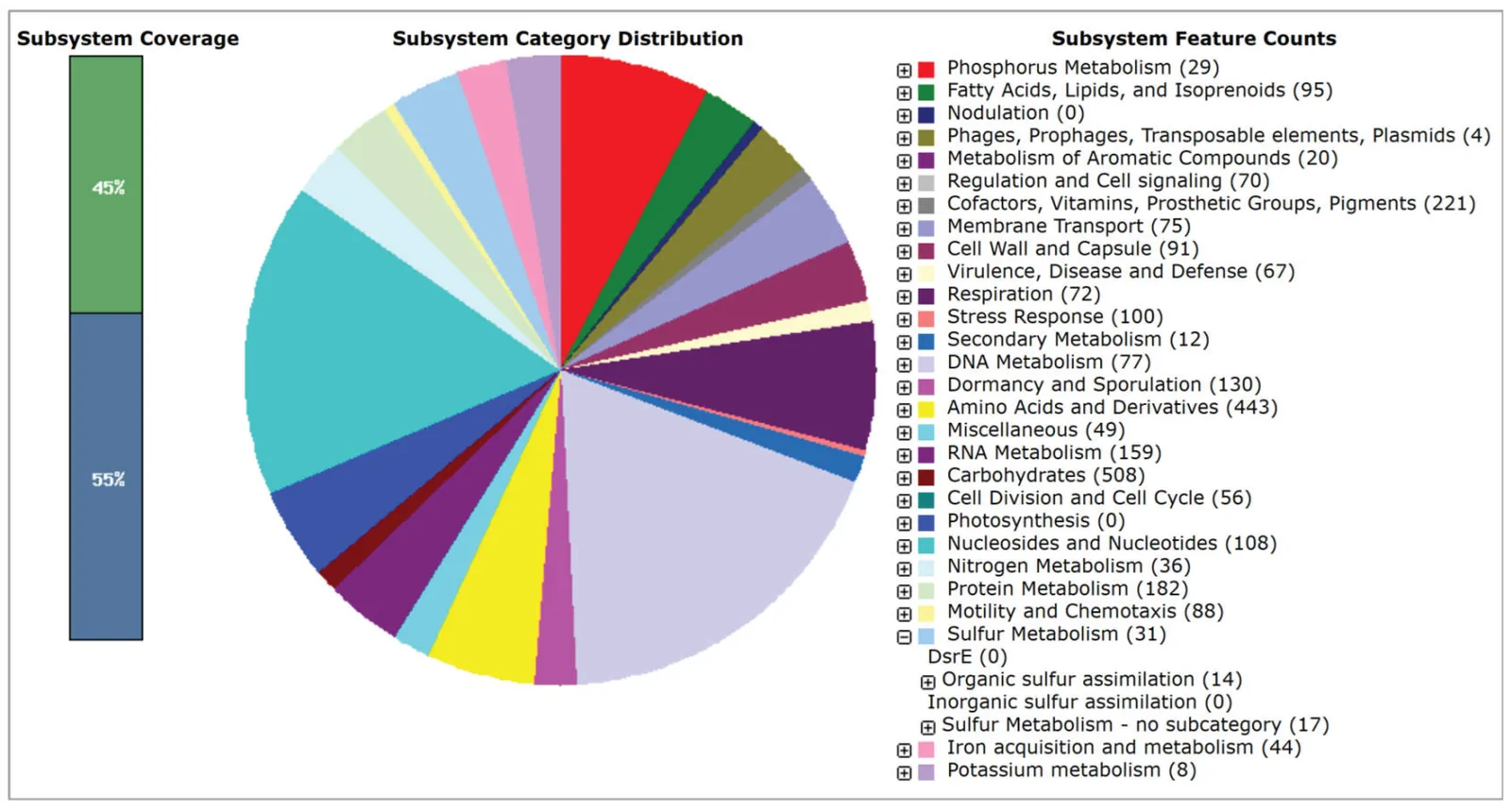
Functional subsystem distribution in strain BLN313 predicted by Classic RAST pipeline (default settings).

Genome annotation of strain BLN313 by the Classic RAST pipeline identified 4373 protein-coding sequences and found a total of 105 RNA genes (Table 2). Functional assignment classified them into 463 subsystems, reflecting the broad metabolic capacity of the strain (Table 2). Carbohydrates (508 ORFs) and amino acids and derivatives metabolism (443 ORFs) were the most prominent subsystems, followed by subsystems related to cofactors, vitamins, prosthetic groups and pigments (221 ORFs), proteins (182 ORFs), RNA (159 ORFs), nucleosides and nucleotides (108), and fatty acids, lipids, and isoprenoids metabolism. Subsystems related to membrane transport (75 ORFs) and stress response (100 ORFs) were also highly represented, implying adaptive potential under environmental stress. Regarding sulfur metabolism, 14 ORFs were classified into assimilation and utilization of organic sulfur compounds or alkanesulfonates, and the other 14 ORFs could not be assigned to any subcategory. In addition, no ORFs similar to those associated with inorganic sulfur assimilation were identified (Figure 5).

### 2.10. CRISPR Elements and Repeat Sequence Analysis

The CRISPR element analysis of the BLN313 genome showed that it lacks CRISPR repeat-spacer arrays. The absence of CRISPR-Cas systems in BLN313, which have been observed in some environmentally adapted *Bacillus* species, might reflect increased genome plasticity. This increased plasticity could facilitate the acquisition of metabolic and stress response genes through horizontal gene transfer, potentially contributing to adaptive traits such as selenium tolerance and transformation [18,19,20].

Repeat sequence analysis revealed only a small number of tandem and interspersed repeats, including transposable elements (Table 4 and Table 5). The low repeat content is consistent with the minimal repetitive fraction estimated during genome size prediction and likely contributed to the assembly of a contiguous circular chromosome. The resulting genome stability may help preserve functionally important selenium- and sulfur-related metabolic pathways.

### 2.11. Species-Level Classification of Strain BLN313 Through Genome-Based Taxonomic and Phylogenomic Analyses

The species-level taxonomic position of strain BLN313 was evaluated by using genome-based criteria by comparing its genome against the most closely related type strains with overall genome-related indices (OGRIs), specifically average nucleotide identity (ANI) and digital DNA–DNA hybridization (dDDH). According to 16S rRNA gene sequence similarity retrieved from the EzBioCloud platform, a total of six type strains including *Bacillus licheniformis* ATCC 14580^T^, *Bacillus haynesii* NRRL B-41327^T^, *Bacillus sonorensis* NBRC 101234^T^, *Bacillus paralicheniformis* KJ-16^T^, *Bacillus glycinifermentans* GO-13^T^, and *Bacillus swezeyi* NRRL B-41294^T^ were selected for overall genome-related index (OGRI) analyses (Table 6). The results show that strain BLN313 shares an ANI value greater than 99% and a dDDH value exceeding 97% with the type strain *Bacillus licheniformis* ATCC 14580^T^. Both values exceed the standard thresholds used to define bacterial species, providing strong evidence that strain BLN313 should be classified as *Bacillus licheniformis*.

**Figure 6:**
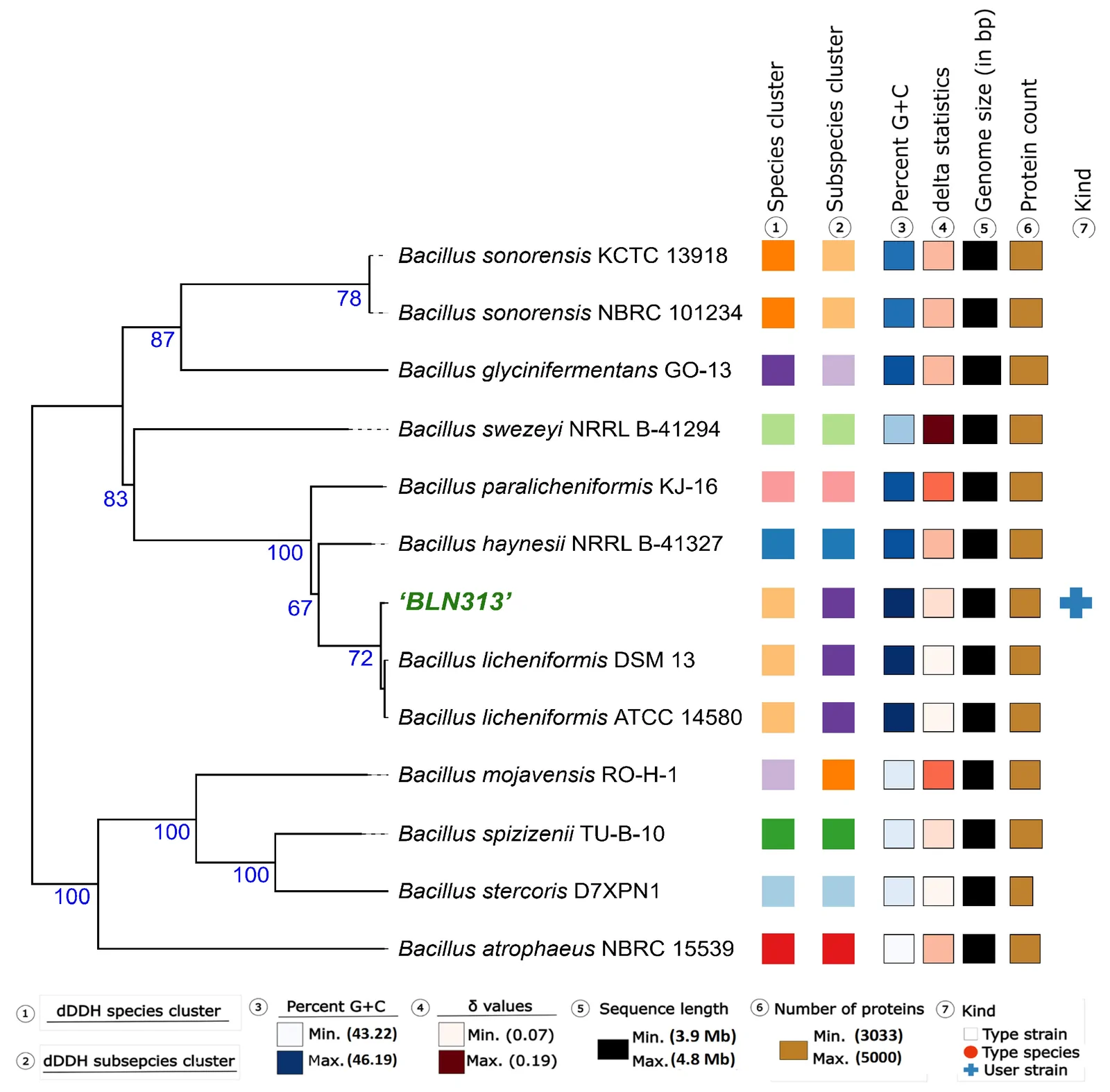
Phylogenomic analysis of strain BLN313 using the Type (Strain) Genome Server (TYGS). The tree was inferred with FastME 2.1.6.1 from GBDP distances calculated from genome sequences. The branch lengths are scaled in terms of GBDP distance formula d5. The numbers above branches are GBDP pseudo-bootstrap support values > 60% from 100 replications, with an average branch support of 84.0%. The tree was rooted at the midpoint. The colored leaf-labels below the tree denote various genome features, with the same color showing species and subspecies within the same taxon.

To further improve the genome-based taxonomic position of strain BLN313, a phylogenomic analysis was conducted through the Type (Strain) Genome Server (TYGS; https://tygs.dsmz.de/ (accessed on 24 April 2025)). The obtained phylogenomic tree (Figure 6) showed that strain BLN313 tightly grouped with type strains *Bacillus licheniformis* DSM 13^T^ and *Bacillus licheniformis* ATCC 14580^T^, with moderate branch support (bootstrap 72; δ = 0.072). The three genomes showed nearly identical genomic G + C content, ranging from 46.19% to 46.28% and a comparable genome size of nearly 4.2 Mb (Figure 6, labels 3, 5). Furthermore, the annotated numbers of protein-encoding genes were highly similar, with 4116 protein-coding sequences identified in strain BLN313 compared with 4179 genes in *B. licheniformis* DSM 13^T^ and 4172 in *B. licheniformis* ATCC 14580^T^ (Figure 6, label 6). These genomic features indicate a high degree of relatedness, greatly supporting the placement of strain BLN313 within the species *Bacillus licheniformis.*

### 2.12. Potential Genes Providing Biogenic Nanoparticle Synthesis Trait in BLN313 and Comparative Analysis with Related Species

Numerous microorganisms are competent in reducing selenium oxyanions, such as selenite (SeO_3_^2−^) and selenate (SeO_4_^2−^), to elemental selenium (Se^0^), often producing nanostructured elements [21,22,23,24,25,26]. This biocatalytic transformation detoxifies soluble selenium species by modifying them into insoluble Se^0^, usually detected as spherical intracellular or extracellular selenium nanoparticles (SeNPs) [12,27,28,29,30]. Selenium reduction in bacteria comprises different redox enzymes and stress response systems and occurs through successive steps including uptake of oxyanion species, their reduction to elemental selenium (Se^0^), and nanoparticle nucleation and assembly [31,32,33,34]. To investigate the genetic determinants of selenium biocatalytic transformation in strain BLN313, the genome sequence of BLN313 and previously reported SeNP-synthesizing bacteria was annotated through the RAST pipeline (Table 7 and Supplementary Table S3). The comparative genomic analysis revealed that BLN313 harbors six copies of *trxB* gene, which encodes thioredoxin reductase, an essential enzyme involved in intracellular redox reactions (Table 7). Thioredoxin-dependent systems support selenite (SeO_3_^2−^) reduction to elemental Se^0^, a key preliminary step in SeNPs formation [32,35,36].

Genes involved in sulfur and methionine metabolism, such as *metE* (5-methyltetrahydrofolate-homocysteine methyltransferase) and *yitJ* (5,10-methylenetetrahydrofolate reductase), were conserved in BLN313 and widely distributed across other Gram-positive and Gram-negative SeNP-synthesizing bacteria (Table 7). These pathways interrelate with selenium metabolism due to their chemical similarity, enabling non-specific incorporation and reduction of selenium oxyanions. Their occurrence facilitates sulfur- and one-carbon-dependent regulation of reduction potential required for SeNP formation [20,36,37]. The BLN313 genome also contains *mmuM* gene, expressing a methyltransferase involved in selenium detoxification through methylated intermediates, regulating SeNP formation (Table 7). Conservation of the *mmuM* gene among *Bacillus* and other SeNP- synthesizing bacteria suggests a highly conserved function in facilitating intracellular selenium stress [27,38]. Genes associated with thiol-dependent redox regulation and stress adaptation, comprising *ypjG* (Bacillithiol biosynthesis deacetylase BshB1), *yojG* (Bacillithiol biosynthesis deacetylase BshB2), and *yjcL* (cystathionine γ-synthase), were also detected in the genomic pool of BLN313 (Table 7). These genes are implicated in bacillithiol and sulfur amino acid metabolism, contributing to redox homeostasis and tolerance to selenium-induced oxidative stress [38]. Comparative genomic analysis showed they are highly conserved in *Bacillus* species but variably distributed across Gram-negative bacteria, indicating lineage-specific reliance on bacillithiol-mediated redox systems. The ypjG and *yojG* genes are primarily linked to glutathione-like redox buffering, whereas *yjcL* is associated with maintaining reduced sulfur pools required for thiol-mediated reactions [37,39]. In BLN313, enrichment of these genes proposes a supporting role in stabilizing redox homeostasis particularly during Se^0^ nucleation.

Furthermore, BLN313 also encodes the *mccA* gene (Table 7), annotated as cystathionine β-synthase-associated c-type cytochrome, showing the possible contribution of electron transfer to selenium reduction, consistent with its presence in other SeNP-producing bacteria [37,40]. In addition, multiple genes encoding catalase were also identified (Table 7), suggesting an efficient oxidative stress defense system crucial for detoxifying reactive oxygen species produced during selenium oxyanion reduction [41,42,43]. Collectively, comparative genomic analysis between BLN313 and well-established SeNP-synthesizing bacteria shows that BLN313 shares a core set of SeNP-related genes with *Bacillus licheniformis*, *Bacillus subtilis*, and *Bacillus mycoides* species while also exhibiting strain-specific gene copy variations. The complete absence or limited representation of several of these genes in lactic acid bacteria and other bacterial species (Table 7) corresponds to their reduced or lower efficiency reported in SeNP synthesis [44,45].

**Figure 7:**
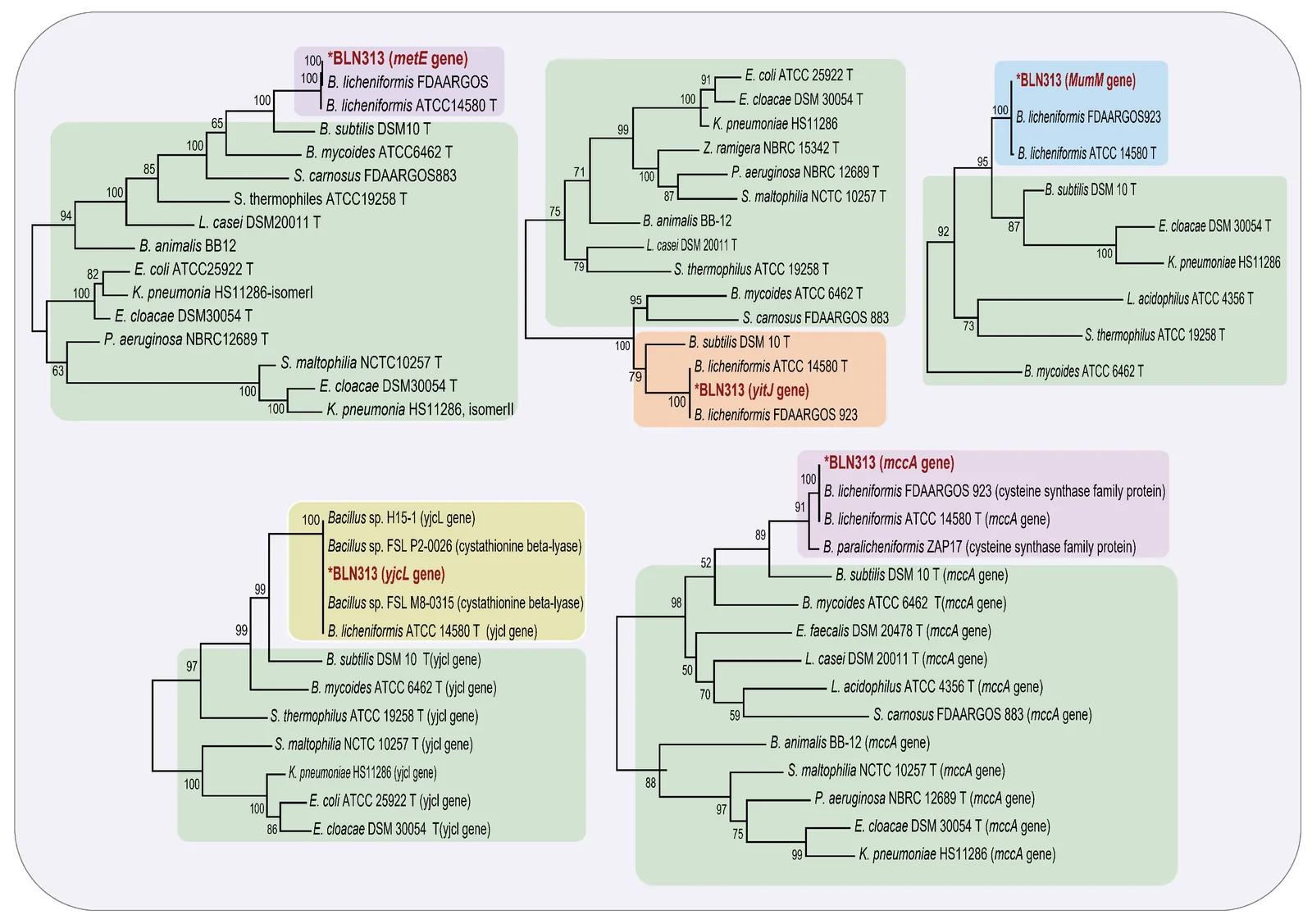
Phylogenetic analysis of key genes associated with selenium nanoparticle (SeNP) biosynthesis in *Bacillus licheniformis* BLN313. The phylogenetic trees were constructed using MEGA X using the Maximum Likelihood method and Tamura–Nei model and branch support was assessed using 100 bootstrap replicates. (*) indicates BLN313 genes used in analysis.

Similarity search analysis of identified selenium-related genes in BLN313 against the NCBI non-redundant database using BLASTn showed high sequence identity (>99%) with homologs from *Bacillus licheniformis* and other *Bacillus* species (Supplementary Table S4). To further elucidate the evolutionary relationship between BLN313 and other SeNP-synthesizing bacteria, particularly for genes with variable distribution (e.g., *metE*, *yitJ*, *yjcL*, *mccA*, and *mmuM*), phylogenetic trees were constructed using MEGA-X based on complete gene sequences retrieved from the NCBI database (Figure 7).

These analyses disclosed that BLN313 genes clustered closely with homologs from *Bacillus licheniformis* and *Bacillus subtilis* while making a secondary cluster with *Bacillus mycoides*, indicating close evolutionary relatedness within the *Bacillus* genus. In contrast, the SeNP-associated genes from other Gram-negative species clustered into completely separate and more distant clades. These findings indicate that strain BLN313 shares a conserved set of selenium-associated gene repertoire with *Bacillus* strains known to be functionally competent. However, the phylogenetic clustering pattern further proposes that selenium-associated genes in the strain BLN313 may have undergone adaptive divergence mediated by environmental selenium stress, rather than purely vertical inheritance [46], which may potentially support efficient SeNP biosynthesis in BLN313.

### 2.13. Identification and Comparative Analysis of Putative Selenium Oxyanion Transporters in BLN313

To determine the potential genes involved in selenium oxyanion uptake in strain BLN313, a comparative genomic analysis was performed. This analysis targeted the well-known and putative anion transporters by comparing them to homologs from well-characterized SeNP-producing bacteria, utilizing BLAST similarity searches and phylogenetic analyses. The results demonstrate that BLN313 encodes several candidate transporters including members of the SulP family (inorganic anion permeases) as well as the Pit family of sodium-dependent phosphate transporters (Table 8). Both of these transporter families are reported to mediate the uptake of oxyanions that are structurally analogous to sulfate and phosphate, such as selenate (SeO_4_^2−^) and selenite (SeO_3_^2−^) [47,48].

The analysis further revealed that strain BLN313 lacks the well-recognized sulfate/selenate ABC transporter operon (CysAWTP) and its related solute binding protein CysP/Sbp, identified in other Gram-negative bacteria such as *Escherichia coli*, *Enterobacter cloacae*, *Pseudomonas aeruginosa*, and *Klebsiella pneumonia* (Table 8). In these species, the CysPTWA transporter system has been experimentally validated to facilitate sulfate and selenate uptake due to their close chemical similarity [47,49]. The absence of this high affinity transporter system in BLN313 suggests its complete reliance on secondary transporters, mainly SulP-family permeases, for selenate acquisition.

**Figure 8:**
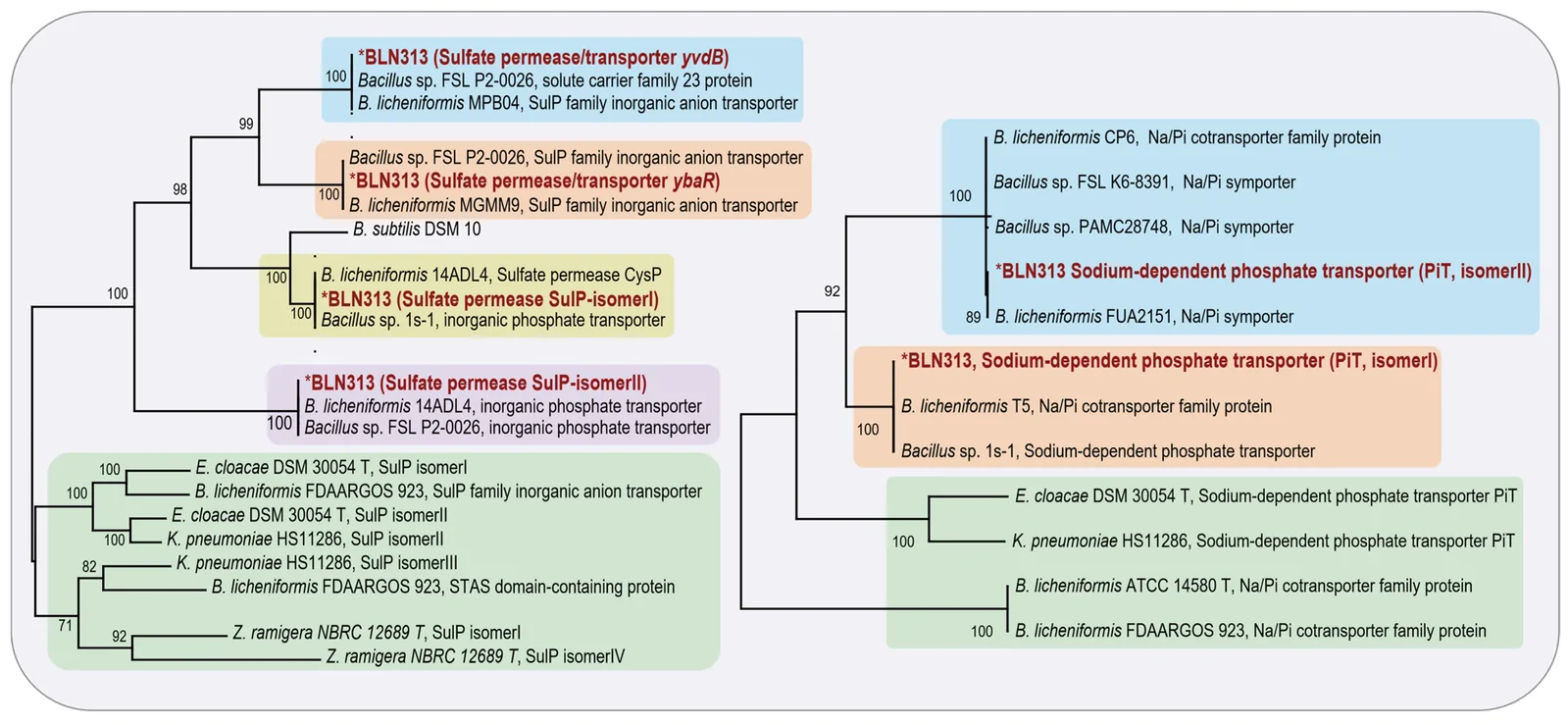
Phylogenetic analysis of sulfate permeases (SulP) and sodium-dependent phosphate transporters (PiT) implicated in selenium oxyanion uptake in *Bacillus licheniformis* BLN313. The phylogenetic trees were constructed using MEGA X using the Maximum Likelihood method and Tamura–Nei model, and branch support was assessed using 100 bootstrap replicates. (*) indicates BLN313 transporters used in analysis.

BLASTn analysis of complete gene sequences of SulP permeases identified in BLN313 genome revealed that they share 100% query coverage and ≥99–100% sequence identity with SulP-family transporters from closely related *Bacillus licheniformis* strains and other *Bacillus* species (Supplementary Table S4). These comprised homologs annotated as SulP-family inorganic anion transporters, sulfate permeases (CysP-like), and solute carrier family 23 proteins. This high degree of sequence similarity indicates that these SulP homologs probably retain similar transport functions. In phylogenetic analysis, BLN313 SulP homologs clustered into distinct, well supported clades (bootstrap value ≥ 98–100) associated with defined functional subgroups, including SulP isomer I, SulP isomer II, and YbaR/YvdB-like sulfate permeases (Figure 8). Particularly, BLN313 SulP homologs formed a close cluster with SulP transporters from *Bacillus licheniformis* and other *Bacillus* species, instead of SulP homologs from more distantly related bacterial species included in the analysis, including *Enterobacter cloacae*, *Klebsiella pneumoniae*, and *Zoogloea ramigera*. This phylogenetic analysis suggests strong evolutionary relatedness of SulP permeases between BLN313 and related *Bacillus* species, consistent with a shared and potentially conserved role in selenium oxyanion uptake.

Similar to SulP homologs, BLAST similarity analysis of PIT homologs, designated as PIT isomer I and PIT isomer II, revealed near complete sequence identity (≥99.7–100%) with Na^+^/phosphate cotransporters from *Bacillus licheniformis* and other *Bacillus* species (Table 7). Phylogenetic analysis positioned BLN313 PIT isomers into two clearly separated clades (bootstrap values ≥ 89–100), corresponding to functionally distinct PIT lineages (Figure 8). Importantly, BLN313 PIT homologs closely grouped with phosphate transporters from *Bacillus licheniformis* and other *Bacillus* species while forming a discrete lineage from PIT homologs of known selenium-transforming bacteria such as *Enterobacter cloacae* and *Klebsiella pneumonia*, in which these transporters have been experimentally related to selenite uptake [50].

The co-occurrence of several SulP isomers and PIT transporters in strain BLN313 corroborates a functionally diverse oxyanion transport mechanism, enabling selenium uptake through both sulfate- and phosphate-analog pathways. A similar transporter-system has been reported in various efficient SeNP-synthesizing bacteria, including *Bacillus subtilis*, *Bacillus licheniformis*, *Bacillus cereus*, *Enterobacter cloacae*, *Pseudomonas putida*, *Klebsiella pneumoniae*, and *Shewanella oneidensis*. In these bacteria, the existence of multiple, partly redundant anion transport systems with broad substrate specificity has been correlated with enhanced selenium oxyanion uptake under variable environmental conditions [11,47,51,52].

The intact sulfate/selenate transport systems were not detected in the genomes of *Lactobacillus*, *Bifidobacterium*, *Streptococcus*, and *Staphylococcus* species included in the analysis, indicating the presence of alternative low-affinity or nonspecific phosphate or anion transporters. The absence of these well-recognized transport systems can be associated with limited selenium oxyanion uptake and a relatively lower efficiency for SeNP biosynthesis in these bacterial species [47,48,51]. Compared with these bacterial species, strain BLN313 harbors a functionally diverse and evolutionarily conserved repertoire of secondary anion transporters, comprising several SulP and Pit isomers, probably facilitating more efficient selenium uptake despite the absence of typical ABC-type sulfate/selenate transport systems.

### 2.14. Proposed Mechanistic Model for Biogenic Selenium Nanoparticle Synthesis in BLN313

**Figure 9:**
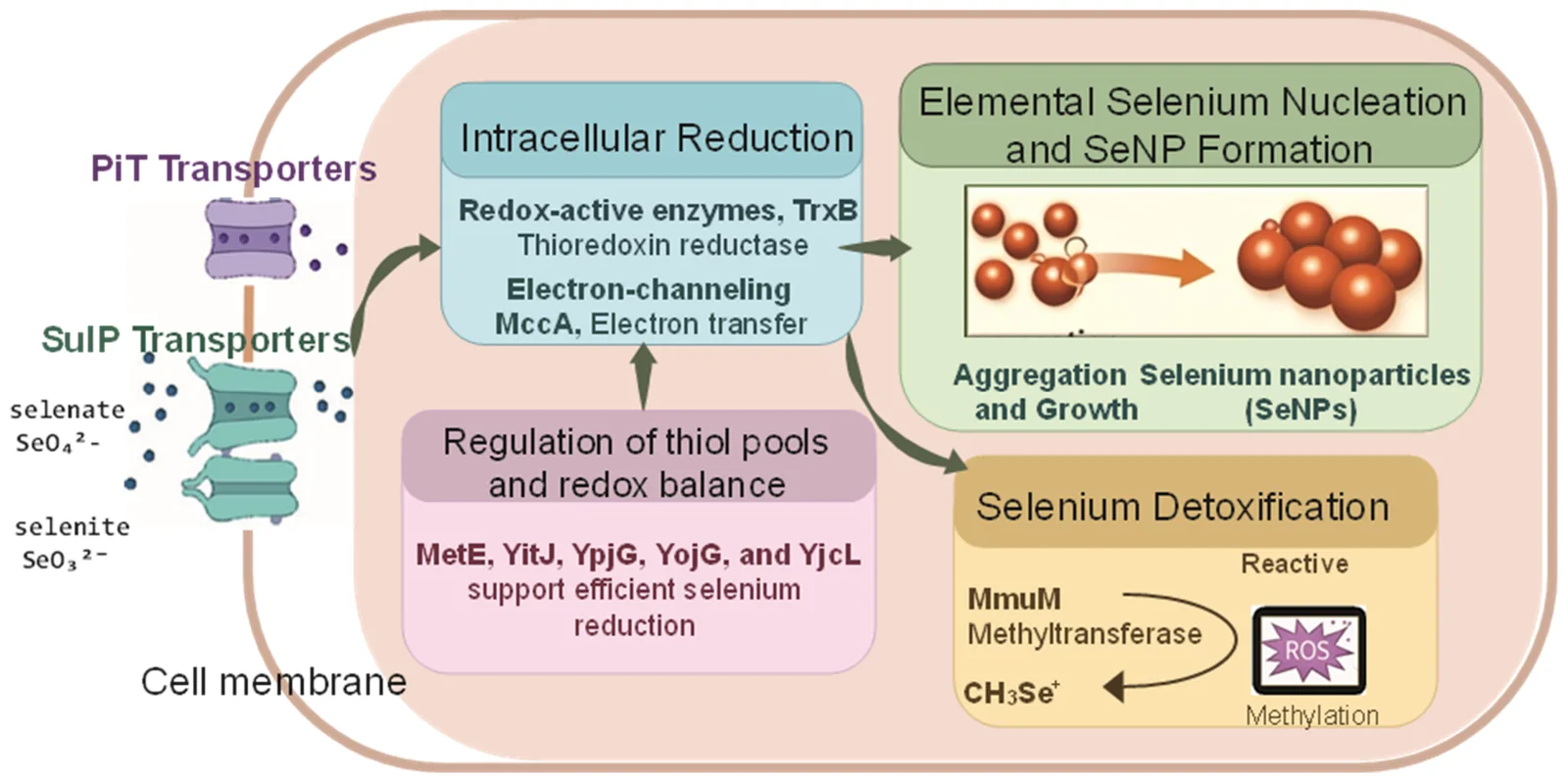
Proposed mechanistic model of biogenic selenium nanoparticle (SeNP) synthesis in *Bacillus licheniformis* BLN313. In this model, the selenate and selenite oxyanions are proposed to enter through SulP-family sulfate permeases and Pit-family sodium-dependent phosphate transporters. Upon entering the bacterial cell, they undergo intracellular reduction through redox-active enzymes such as thioredoxin reductase (TrxB), a major enzyme with associated electron-transfer proteins (e.g., MccA) supplying the reducing equivalents. Moreover, the conserved set of genes including *metE*, *yitJ*, *ypjG*, *yojG*, and *yjcL* facilitate this reduction process by regulating the thiol pools and redox balance. Following reduction, the reduced selenium intermediates nucleate to form elemental selenium (Se^0^), followed by growth and aggregation into SeNPs. Concurrently, MmuM mediates the methylation of the reduced selenium intermediates, contributing to selenium detoxification and modulation of reactive oxygen species. Catalases are also supposed to be involved in alleviating oxidative stress produced during selenium oxyanion reduction. This model reflects patterns inferred from comparative genomic and phylogenetic analysis and has not yet been functionally validated.

Through comparative genomic analysis of selenium-associated genes combined with BLAST similarity searches and phylogenetic analysis, we propose a mechanistic model for biogenic SeNP synthesis in BLN313 that links selenium acquisition, intracellular reduction, redox homeostasis, and nanoparticle formation (Figure 9). In this proposed model, first, selenium oxyanions (selenate/selenite) are internalized through secondary anion transport systems, mainly SulP-family sulfate permeases and Pit-family sodium-dependent phosphate transporters, which possibly drive selenium uptake owing to the structural similarity between selenium oxyanions and sulfate/phosphate.

Subsequently, selenium oxyanions undergo sequential intracellular reduction regulated by redox-active enzymes. Thioredoxin reductase (TrxB) plays a vital role in this process, providing reducing equivalents through the thioredoxin system. Complementary electron-transfer components, comprising MccA and other oxidoreductases, are putatively involved in channeling electron flow toward selenium oxyanions. Particularly, the conserved set of *metE*, *yitJ*, *ypjG*, *yojG*, and *yjcL* genes detected in the BLN313 genome are proposed to facilitate this reduction process. These genes encode enzymes related to sulfur metabolism and redox regulation and are possibly implicated in regulating reduced intracellular thiol pools and redox balance, thereby indirectly supporting efficient selenium oxyanion reduction.

Following the accumulation of reduced selenium intermediates, elemental selenium (Se^0^) is produced, which proceeds through intracellular nucleation, followed by growth and aggregation into selenium nanoparticles. This process is supposed to take place in association with cytoplasmic or membrane-associated components, in accordance with models of intracellular SeNP formation reported in other bacteria [46,53]. At the same time, methyltransferase MmuM mediates the methylation of reactive selenium intermediates (reactive oxygen species, ROS), providing an additional detoxification route, preventing excessive selenium accumulation and alleviating oxidative stress produced during selenium reduction. In addition, multiple genes encoding catalase were also identified, suggesting an efficient oxidative stress defense system crucial for detoxifying reactive oxygen species produced during selenium oxyanion reduction. These enzymes are known to facilitate both cellular protection and SeNPs’ stability. On the whole, this integrated pathway is in agreement with established models of bacterial selenium metabolism, where SeNP formation corresponds to both the detoxification strategy as well as the downstream outcome of cellular redox activity [53].

## 3. Discussion

Bacterial synthesis of selenium nanoparticles represents a biocompatible and environmentally sustainable alternative to conventional chemical methods. Interest in this process has increased rapidly owing to its prospective environmental and biomedical applications [42,54,55,56,57,58,59]. Realization of this potential, however, largely depends on the availability of strains with a well-characterized genomic basis for selenium handling, which can be predicted or engineered. This study aims to address this knowledge gap for *Bacillus licheniformis* BLN313.

BLN313 tolerated selenite at concentrations well above those typically reported as inhibitory, converting 88.8% of the supplied selenite to Se^0^ at 500 µg/mL. This value is comparable to the 99.1% conversion reported for *Bacillus paramycoides* 24522 [60], 76.4% reported for *B. paralicheniformis* Y4 [35], and the efficiencies described for other selenite-reducing *Bacillus* strains under optimized conditions [61,62]. More informative than the peak conversion value, however, is the divergence between conversion efficiency and viability; reduction decreased from 88.8% at 0.5 mg/mL to 25.0% at 1.0 mg/mL and 3.0% at 5.0 mg/mL, while the culture remained metabolically active across the entire concentration range. Tolerance and detoxification therefore constitute distinct traits in this strain. General stress responses maintain cell viability well beyond the point at which the reductive system, a finite NADPH-dependent enzymatic pathway, can match the substrate load. This distinction is critical for scale-up, because the selenite concentration that maximizes biomass is not the same as that which maximizes SeNP yield.

The physicochemical data should be interpreted collectively for a comprehensive understanding. TEM revealed a polydisperse population with a mean diameter of 190 ± 52 nm, whereas DLS indicated a smaller Z-average diameter of 165.8 ± 0.4 nm for particles in suspension. This size range aligns with those reported for other biogenic SeNP preparations [57,63,64,65]. The discrepancy between TEM and DLS measurements is anticipated, as these techniques evaluate particles under different physical conditions and employ distinct principles for size determination. The moderately negative zeta potential (−20.8 ± 0.2 mV), along with FTIR and XPS evidence of surface-associated biomolecules, implies that biomolecular capping may enhance colloidal stabilization in conjunction with electrostatic interactions [66]. XRD further identified trigonal Se^0^ reflections with an apparent crystallinity of approximately 29% and a predominant diffuse/noncrystalline contribution. Consequently, BLN313-SeNPs are more accurately characterized as containing crystalline trigonal Se^0^ domains within a significant diffuse/noncrystalline component, rather than as entirely crystalline particles.

The UHPLC-ESI-Orbitrap-MS metabolite screening provides a molecular fingerprint that enhances the current understanding. Muramic acid and UDP-N-acetylmuraminate, both linked to peptidoglycan, were found to be enriched on the nanoparticle surface, along with metabolites related to amino acids and sulfur, such as L-lysine, pipecolic acid, 5′-methylthioadenosine and L-phenylalanine. The presence of these peptidoglycan-associated metabolites indicates a contribution from cell-envelope-derived material to the biomolecular layer associated with the nanoparticles, corroborating the FTIR and XPS evidence of surface-associated biomolecules. This interpretation aligns with previous findings that the composition of biogenic SeNP capping layers differs among bacterial producers [67].

The preliminary antibacterial and anticancer assays merit discussion because of the distinct response patterns observed. The activity was more pronounced against *M. luteus* compared to *K. pneumoniae*, aligning with the known permeability differences between Gram-positive and Gram-negative bacterial envelopes. Specifically, the outer membrane of Gram-negative bacteria restricts the entry of both small molecules and particulate agents [68,69]. Additionally, agar-based assays tend to under-report nanoparticle activity due to limited diffusion through the medium [70]. The minimum inhibitory concentration (MIC) values obtained in this study fall within the range previously reported for biogenic SeNPs [71,72,73]. Notably, sodium selenite alone did not exhibit inhibition under identical conditions, indicating that the observed activity is associated with the particles, consistent with the reactive oxygen species (ROS)-mediated membrane damage and cytoplasmic leakage described as antibacterial mechanisms for biogenic SeNPs [74]. The anticancer findings are particularly noteworthy when considered alongside the selectivity data. BLN313-SeNPs reduced MCF-7 cell viability with an IC50 of 2.7 µg/mL, which is lower than the 17.6 µg/mL reported for *Bacillus coagulans*-derived SeNPs [75] and other biogenic preparations active in the tens of µg/mL range [76,77]. In contrast, MCF-10A normal mammary epithelial cells maintained over 97% viability across the same 1–1,000 µg/mL concentration range. Such selective toxicity has been linked to ROS-mediated necroptosis via TNF activation, a pathway preferentially activated in cancer cells [78]. However, we refrain from asserting that this mechanism is operative in this context, as cellular uptake and apoptotic or necrotic markers were not assessed, leaving the explanation based on precedent rather than direct evidence from this system.

Genome-based taxonomy has classified BLN313 within *Bacillus licheniformis*, supported by ANI and dDDH values exceeding accepted species thresholds [79], with phylogenomic placement corroborated by TYGS [80]. The genome size (4.11 Mb) and GC content (46.3%) align with sequenced conspecifics such as MCC 2514 (4.23 Mb; 46.2% GC) [81]. The absence of CRISPR-Cas systems and low repetitive-sequence content suggest, but do not definitively establish, increased genome plasticity. In *Bacillus cereus* group isolates, the loss of CRISPR-Cas has been associated with enhanced horizontal gene transfer and mobile element acquisition [82]. A similar reduction in defense against foreign DNA may have enabled BLN313 to accumulate the diverse selenium-handling genes identified here. This hypothesis aligns with the observed genome architecture but does not establish a causal link between CRISPR-Cas loss and the selenium phenotype. Proper testing would necessitate comparative genomics across a defined panel of strains with and without CRISPR-Cas systems, which was beyond the scope of this study.

The reconstruction of the likely pathway from selenium oxyanion to nanoparticle was based entirely on comparative genomics, necessitating clarity regarding the capabilities and limitations of this approach. BLN313 possesses multiple SulP and Pit transporter homologs and lacks the canonical CysAWTP ABC transporter system found in many Gram-negative selenate reducers, suggesting that uptake occurs through these secondary routes rather than the primary ABC system [47,49]. Downstream, an increased *trxB* copy number, along with *metE*, *yitJ*, *ypjG*, *yojG*, *yjcL*, and *mccA*, indicates a thioredoxin-coupled reduction pathway integrated with sulfur metabolism, consistent with thioredoxin-dependent routes reported in selenium-reducing bacteria [83,84,85]. Additionally, multiple catalase genes, together with *mmuM*, suggest parallel routes for oxidative and selenium-intermediate detoxification. Collectively, these genes propose a plausible pathway of uptake, reduction, and detoxification. However, inferring a pathway from gene content does not equate to demonstrating its operation in BLN313. Due to the absence of an established transformation system for BLN313 and the lack of transporter-specific inhibitors, genetic knockout and chemical inhibition assays were not feasible. Consequently, transcriptomic profiling and targeted mutagenesis represent the logical next steps to transition this model from plausible to confirmed.

Two recent studies have contributed to this field from distinct perspectives. The first study, involving transcriptomic analysis in *Bacillus subtilis* 168, identified PutC, GabD, and the CysJI complex as contributors to selenite reduction [11]. The second study focused on fermentation optimization in *Bacillus licheniformis* F1, demonstrating the significant impact of culture conditions on SeNP yield and characteristics [9]. However, neither study established a connection between a confirmed SeNP-producing phenotype and a comprehensive genome-scale account of the transport, reduction, and detoxification genes involved. This study addresses that gap by providing a validated producer strain with a mapped selenium-handling gene repertoire. This mapping offers concrete targets for future transcriptomic and mutagenesis research, as opposed to an uncharted genome. The potential utility of BLN313 for biomedical or environmental applications will depend on subsequent research confirming the functional roles of the candidate genes identified in this study.

## 4. Materials and Methods

### 4.1. Chemicals and Reagents

All chemicals and reagents were of analytical, reagent, or cell-culture grade. Inorganic salts and reagents including sodium selenite (Na_2_SeO_3_; ≥98.0%), NaOH (≥96.0%), NaCl (≥99.5%), and HCl (≥37%) were purchased from Sinopharm Chemical Reagent Co., Ltd. (Shanghai, China). Tryptone, agar powder, and yeast extract (LP0021) were obtained from Beijing Aoboxing Biotechnology Co., Ltd. (Beijing, China), Beijing Kangbeisi Technology Co., Ltd. (Beijing, China), and Oxoid (Basingstoke, UK), respectively. Other reagents, solvents and cell-culture supplements were obtained from Solarbio (Beijing, China), Sigma-Aldrich (St. Louis, MO, USA), and Gibco (Thermo Fisher Scientific, Waltham, MA, USA). A complete list of chemicals, including formulas, grade/purity, and suppliers, is provided in Supplementary Table S1.

### 4.2. Strain Activation and Culture Conditions

The bacterial strain BLN313, previously reported as BSN313, was isolated from indigenous Chinese Jiuqu (Linyi, Shandong province, China). It was chosen for its Se resistance and relatively high Se enrichment potential, and the detailed isolation procedure has been described earlier [61]. The strain was maintained in 20% (*v*/*v*) glycerol suspension at −80 °C. For culture activation, the single colonies were obtained by slowly thawing the frozen stocks on ice followed by streaking and incubating onto Luria–Bertani (LB) agar plates at 37 °C for 12 h. LB media was formulated by dissolving yeast extract (0.5 g), tryptone (1 g), and NaCl (1 g) in distilled water (100 mL). In contrast, solidified LB agar was constituted by adding agar powder (1.2 g/mL of media) followed by thermal sterilization in an autoclave at 121°C for 15 min. An actively growing culture was achieved by inoculating a single colony into LB broth (4 mL) in a 10 mL culture tube with constant agitation (220 rpm) at a controlled temperature of 37 °C for 12 h.

### 4.3. Sodium Selenite Stress and Induction of Nanoparticle Synthesis

To investigate the response of strain BLN313 to sodium selenite (Na_2_SeO_3_) stress, 1 mL of exponentially growing culture was transferred into Erlenmeyer flasks (250 mL) containing LB media (100 mL) supplemented with different concentrations (100 ug/mL to 10 mg/mL) of analytical-grade Na_2_SeO_3_. The stock solution of Na_2_SeO_3_ (50 mg/mL) was formulated in accordance with the protocol delineated by Ullah et al., 2020 [61]. These flasks containing varying concentrations of Na_2_SeO_3_ were placed in an incubator with constant agitation (220 rpm) at 37 °C for 40 h. To assess the bacterial growth, absorbance at a wavelength of 600 nm (OD_600_) was recorded every 4 h. The chromogenic shift of medium to red color in the flask indicated Se^4+^ reduction to elemental Se^0^, preliminary confirming the formation of biogenic selenium nanoparticles. All experimental procedures were conducted in triplicate.

### 4.4. Determination of Total Selenium (Se^0^) Concentration by LC-AFS

Total concentration of reduced elemental selenium (Se^0^) in the BLN313 cells was determined by using Liquid Chromatography–Atomic Fluorescence Spectrophotometry (LC-AFS). Sample preparation and acid digestion were carried out following the protocol as delineated by Ullah et al., 2020, with minor modifications [61]. Briefly, the cell biomass was collected by centrifuging the bacterial culture at 9000 rpm for 10 min. The resulting pellets were washed twice with 5 mL of sterile deionized water to eliminate residual extracellular selenite. These pellets were dispersed in deionized water (1 mL) and then transferred to 25 mL microwave digestion tubes. To digest the samples, 4 mL of concentrated HNO_3_ was used at 165 °C for 120 min. After cooling, concentrated HCl (4 mL) was added into each sample followed by heating at 165 °C until the remaining volume was reduced. The resulting digests were cooled down and transferred quantitatively to 50 mL volumetric flasks, diluted with 5% HCl, and adjusted to the final volume to be subjected to LC-AFS analysis through hydrogen generation. Each sample was prepared and analyzed in triplicate.

### 4.5. Purification and Physiochemical Characterization of Biogenic Selenium Nanoparticles

Selenium nanoparticles synthesized by BLN313 at a 500 µg/mL concentration of sodium selenite salt (Na_2_SeO_3_) in three independent biological replicates (*n* = 3) were purified and characterized. Initially, they were cultivated in LB broth at an optimal temperature and constant agitation (37 °C, 220 rpm) without salt addition. After 4 h of initial incubation, the bacterial culture was supplemented with 500 µg/mL of Na_2_SeO_3_ followed by 16 h of incubation under these conditions. According to Sonkusre et al.’s 2014 [13] method, extraction and purification of SeNPs was conducted with slight optimizations. For this, the bacterial cell culture was centrifuged at 6000× *g* for 10 min followed by washing twice with sterile water and subsequently resuspending the cell pellet in 20 mL of sterile water. Furthermore, 120 µL of lysozyme solution ((100 mg/mL)) was added into the cell pellet and incubated at 37 °C for approximately 3 h. Cell disruption was performed via ultrasonication, and the resulting lysate was washed four times. Each time included centrifugation at 15,000× *g* for 10 min with 1.5 M Tris HCl (pH 8.3) supplemented with 1% SDS. The resulting pellet obtained was resuspended again in 16 mL of sterile water, and phase separation was achieved by adding 1-octanol. The aqueous phase comprising SeNPs was centrifuged at 16,000× *g*, followed by sequential washing with the chloroform, absolute ethanol, 70% ethanol, and sterile water. Following a 24 h incubation at 4 °C, the produced SeNPs were accumulated via centrifugation at 16,000× *g*, purified, freeze-dried, and used for subsequent characterization [13].

#### 4.5.1. Scanning Electron Microscopy (SEM)–Energy-Dispersive X-Ray Spectroscopy (EDS)

SEM was used to determine the surface morphology and size of SeNPs. For this analysis, an aliquot of the SeNP suspension was transferred onto a silicon wafer, air-dried to form a small aliquot, and attached to an SEM stub using conductive carbon tape. The sample was coated with a thin layer of carbon, and the images were taken using a ZEISS Sigma 36 field-emission scanning electron microscope (ZEISS, Jena, Germany) operating at an accelerating voltage between 5 and 10 kV. Elemental analysis was conducted by EDS. An Oxford XplorER 30 detector (Oxford Instruments, High Wycombe, UK) coupled to the SEM was used. EDS spectra were obtained from several regions of the sample to determine the elemental composition. Selenium identification and determination of nanoparticles was based on its characteristic Lα emission peak at approximately 1.37 keV.

#### 4.5.2. Transmission Electron Microscopy (TEM)

To determine the nanoparticle morphology and size distribution, TEM was used. Sample preparation was followed by depositing a 10 uL aliquot of the SeNP suspension onto a carbon-coated copper grid that was allowed to dry at 25 °C. At an accelerating voltage of 200 kV, various images of the SeNP suspension were acquired by using the Thermo Fisher Scientific-Talos F200X. Particle size was assessed by using Nano Measurer 1.2 (Fudan University, Shanghai, China) with results reported as the mean ± standard deviation.

#### 4.5.3. Dynamic Light Scattering (DLS) and Zeta Potential Analysis

The hydrodynamic size and surface charge of BLN313-SeNPs were assessed using a Malvern Zetasizer. Purified SeNP suspensions were dispersed in distilled water and transferred to disposable sizing cuvettes for DLS analysis; zeta potential was measured using clear disposable zeta cells. All measurements were conducted at 25 °C in triplicate, and the results are expressed as the mean ± standard deviation of the Z-average diameter, polydispersity index (PDI), and zeta potential.

#### 4.5.4. X-Ray Diffraction (XRD)

XRD was employed to identify the crystalline nature of the nanoparticles. To analyze the crystallinity, lyophilized SeNPs were converted into dried powder. The resulting powder was subjected to X-ray diffraction through a Cu Ka source (λ = 1.5406 Å). The diffraction pattern was recorded at a 2θ range of 10−80°, with a step size of 0.02° and a counting time of 1.0 s per step.

#### 4.5.5. Fourier-Transform Infrared (FTIR) Analysis

FTIR was used to determine the surface chemistry of nanoparticles. Briefly, the lyophilized SeNP powder was homogenized with KBr (1:100) followed by compressing it into a pellet. A Thermo Scientific Nicolet 6700 spectrophotometer (Thermo Fisher Scientific, Madison, WI, USA) was employed to obtain spectra from a 4000 to 400 cm^−1^ range at a 4 cm^−1^ resolution with an average of 32 scans. The peaks in the spectra were interpreted to determine the characteristic functional groups indicative of the SeNPs’ surface. To distinguish SeNP-associated FTIR bands from background bacterial biomolecular signals, a blank bacterial sample prepared without sodium selenite was analyzed under identical conditions and included in the Supplementary Materials.

#### 4.5.6. X-Ray Photoelectron Spectroscopy (XPS)

XPS was performed using a Thermo Scientific Nexsa G2 system (Thermo Fisher Scientific, East Grinstead, UK) to examine the surface elemental composition and chemical state of BLN313-SeNPs. Spectra were acquired using a monochromatic Al Kα X-ray source at a photoelectron collection angle of 60° relative to the sample surface, with an analysis area of approximately 200 μm^2^. Binding energies were calibrated using the C–C component of the C 1s peak at 284.8 eV. Survey spectra were used to identify surface elements based on characteristic binding energy positions.

### 4.6. Metabolite Profiling by UHPLC-ESI-Orbitrap-MS

For the extraction of metabolites, 30 mg of the sample was subjected to extraction with 1 mL of pre-cooled 50% methanol. This process involved glass-bead homogenization, repeated freeze–thaw and grinding cycles, and incubation at −20 °C. Following centrifugation, the supernatant was vacuum-dried, reconstituted in 150 µL of 50% methanol, centrifuged once more, and filtered through a 0.22 µm membrane. A final-wash supernatant, processed identically, was used as the control. Chromatographic separation was performed using a Vanquish Flex UHPLC system (Thermo Fisher Scientific, Germering, Germany) equipped with an ACQUITY UPLC HSS T3 column (2.1 × 100 mm, 1.7 µm). The mobile phases consisted of water containing 0.1% formic acid and acetonitrile, with a flow rate of 0.3 mL/min at 50 °C. Metabolite detection was conducted using a Q Exactive hybrid quadrupole-Orbitrap mass spectrometer (Thermo Fisher Scientific, Bremen, Germany) with HESI-II, operating in both positive and negative ionization modes, employing full-scan/dd-MS^2^ acquisition. The raw data were processed using Progenesis QI 3.0, and metabolites were putatively annotated against HMDB and LIPID MAPS, based on accurate mass, MS^2^ fragmentation, and isotope patterns.

### 4.7. Antibacterial Efficacy of Biogenic SeNPs

The antibacterial efficacy of biogenic selenium nanoparticles (SeNPs) synthesized from *Bacillus licheniformis* BLN313 was evaluated against *M. luteus* (Gram-positive) and *K. pneumoniae* (Gram-negative) using disk diffusion and broth microdilution assays, following the standard procedures outlined by the Clinical and Laboratory Standards Institute (Clinical and Laboratory Standards Institute, 2024a; Clinical and Laboratory Standards Institute, 2024b) [86,87]. In the disk diffusion assay, LB agar plates inoculated with bacterial suspensions (0.5 McFarland standard) were tested with ciprofloxacin (positive control), SeNPs (test sample), sodium selenite (ionic control), and sterile water (negative control). Following a 24 h incubation period at 37 °C, the zones of inhibition were measured along two perpendicular axes and averaged from triplicate assays (*n* = 3. The minimum inhibitory concentration was ascertained through broth microdilution (ranging from 15.6 to 500 µg/mL), with SeNP-only blanks included. The minimum bactericidal concentration was confirmed via subculturing. MIC and MBC were recorded as broth microdilution endpoint values. For antimicrobial testing, SeNPs were suspended in sterile water, while sodium selenite (500 µg/mL) was utilized solely for SeNP biosynthesis. 

### 4.8. Cell Culture and MTT Cytotoxicity Assay

Human MCF-7 breast cancer cells (ER^+^, PR^+^; NIBGE Cell Culture Collection) were maintained in Dulbecco’s Modified Eagle’s Medium (DMEM) supplemented with 10% heat-inactivated fetal bovine serum (FBS), 1% penicillin–streptomycin (100 IU/mL penicillin and 100 µg/mL streptomycin), 2 mM L-glutamine, and 1% non-essential amino acids (NEAAs) at 37 °C in a humidified atmosphere containing 5% CO_2_. The cells underwent subculturing every 3–5 days using 0.25% trypsin–EDTA at a 1:3 split ratio. For cytotoxicity assessment, cells were seeded in 96-well plates at a density of 1000 cells per well and allowed to adhere overnight. Subsequently, the medium was replaced, and cells were exposed to SeNPs at concentrations ranging from 1 to 1000 µg/mL in complete medium (final volume of 100 µL per well) for 72 h, with untreated cells serving as controls. SeNP stocks were prepared in serum-free medium and briefly sonicated prior to serial dilution to ensure uniform dispersion. Post-treatment, morphological changes were examined using an inverted phase-contrast microscope (IMT-2; Olympus, Tokyo, Japan). Cell viability was assessed via the MTT assay by adding MTT solution (10 µL; 12 mM; Life Technologies, Carlsbad, CA, USA) to each well and incubating for 4 h at 37 °C. Following incubation, the medium was completely removed, and formazan crystals were solubilized in 100 µL of undiluted DMSO per well (final concentration 100% *v*/*v* at this terminal solubilisation step), applied identically to all wells including untreated controls, prior to absorbance measurement at 570 nm using a Synergy H1 microplate reader (BioTek, Winooski, VT, USA). Viability (%) was calculated as (A_570_ treatment/A_570_ control) × 100. All treatments were conducted in triplicate wells, and experiments were independently repeated three times (*n* = 3). IC_50_ values were determined through nonlinear regression using GraphPad Prism (GraphPad Software, version 9.0, San Diego, CA, USA). Biocompatibility was further confirmed in MCF-10A normal mammary epithelial cells using the MTT assay performed under the same experimental conditions described above, following standard MTT methodology [88].

### 4.9. Whole-Genome Sequencing and Assembly Statistics of Bacillus licheniformis BLN313

BLN313 genomic DNA was sequenced employing a hybrid strategy integrating short-read Illumina NovaSeq and long-read PacBio Sequel technologies. For Illumina sequencing, a 2 × 150 bp library with an average insert size of 400 bp was prepared, while PacBio sequencing employed a long-insert library of approximately 10 kb. Raw Illumina reads were subjected to initial quality assessment using FastQC, followed by adapter removal and quality filtering with fastp. High-quality filtered reads were subsequently used for error correction and polishing of the genome assembly. PacBio long reads data assembly was performed de novo by using Unicycler (v0.4.8) and Flye (v2.9.1). The resulting assembly was further refined by iterative polishing with Illumina data using Pilon (v1.18). Genome quality and potential contamination were evaluated using the ContEst16S tool available through EzBioCloud (https://www.ezbiocloud.net/, accessed on 18 April 2025). Genome coverage was calculated based on the filtered Illumina sequencing dataset.

### 4.10. Non-Coding RNA, CRISPR, and Repeat Sequence Analysis

The genome of BLN313 was analyzed for non-coding RNAs (ncRNAs) by using an integrated set of specialized bioinformatic tools. Transfer RNA genes, along with their predicted secondary structures, were identified with high sensitivity using tRNAscan-SE. Ribosomal RNA genes, including 5S, 16S, and 23S rRNA genes, were annotated using Barrnap. Additional regulatory ncRNAs were detected through sequence similarity searches against the Rfam database. The existence of CRISPR (clustered regularly interspaced short palindromic repeat) loci, comprising both direct repeats and spacer sequences, was examined using CRISPR Finder. Repeat sequences across the genome were identified using both homology-based and de novo approaches. Interspersed repeats and transposable elements were detected with RepeatMasker (v4.0.5), through comparison with the Repbase database. Tandem repeats, including both microsatellites and minisatellites, were characterized using Tandem Repeats Finder (TRF, v4.10.0).

### 4.11. Functional Genome Annotation and Genome-Resolved Taxonomic Analysis

The Rapid Annotation using Subsystem Technology server (Classic RAST, version 2.0) under standard parameters was used to perform functional genome annotation of strain BLN313 [89]. Initially, the genome-based taxonomic classification of strain BLN313 was assessed through the 16S rRNA gene sequence similarity analysis against validated type strains available through the EzBioCloud platform [90] Closely related taxa exhibiting the highest degree of 16S rRNA gene sequence similarity were subsequently subjected to whole-genome relatedness analyses. Genome relatedness was evaluated by digital DNA–DNA hybridization (dDDH) values using the Genome-to-Genome Distance Calculator (GGDC; version 2.1) [91]. Average nucleotide identity (ANI) was measured using the ANI calculator provided by the Kostas Lab webserver. Phylogenomic analysis was conducted through the Type (Strain) Genome Server (TYGS) (https://tygs.dsmz.de/ (accessed on 24 April 2025)) [80]. The assembled genome sequence in FASTA format was uploaded using the default parameters, and a genome-scale phylogenetic tree was generated based on whole-genome sequence comparisons.

### 4.12. Comparative Genomic Analysis

For comparative genomic analysis of genes related to selenium uptake, redox metabolism, and stress response, the whole-genome sequences of closely related type strains, along with representative selenium nanoparticle (SeNP)-synthesizing bacteria, were retrieved from the NCBI GenBank repository (https://www.ncbi.nlm.nih.gov, accessed on 18 April 2025). All genome sequences were subsequently annotated using the Rapid Annotation using Subsystem Technology (RAST) server (https://rast.nmpdr.org/, accessed on 18 April 2025) under standard parameters, allowing for uniform annotation and reliable subsystem-level comparisons across strains. Owing to the close phylogenetic relatedness, *Bacillus licheniformis* ATCC 14580^T^ (CP140161.1) and *Bacillus subtilis* DSM 10^T^ (CP120681.1) were designated as reference genomes. For a broader comparative analysis, additional *Bacillus* and non-*Bacillus* taxa were also included, representing both the Gram-positive and Gram-negative bacteria previously reported to exhibit selenium transformation capabilities. These bacterial taxa included *Bacillus mycoides* ATCC 6462^T^ (CP009692.1), *Lactobacillus acidophilus* ATCC 4356^T^ (CP139575.1), *Lactobacillus casei* DSM 20011^T^ (AP012544.1), *Escherichia coli* ATCC 25922^T^ (ASHD01000001.1), *Bifidobacterium animalis* BB-12 (ASM2524v2), *Enterococcus faecalis* DSM 20478^T^ (CP118962.1), *Enterobacter cloacae* DSM 30054^T^ (CP056776.1), *Staphylococcus carnosus* FDAARGOS_883 (CP065711.1), *Pseudomonas aeruginosa* NBRC 12689^T^ (CP012001.1), *Zoogloea ramigera* NBRC 15342^T^ (BJNV01000001.1), *Klebsiella pneumoniae* HS11286 (CP003200.1), *Streptococcus thermophilus* ATCC 19258^T^ (CP038020.1), and *Stenotrophomonas maltophilia* NCTC 10257^T^ (LT906480.1). A detailed list of SeNP-associated genes identified across these genomes is provided in Supplementary Table S3.

### 4.13. Statistical Analysis

Data are presented as the mean ± SD of three independent biological replicates (*n* = 3). Normality and homogeneity of variance were confirmed using Shapiro–Wilk and Brown–Forsythe tests. Group differences were analyzed by one-way ANOVA with Tukey’s post hoc test (all pairwise) or Dunnett’s test (versus control), and bacterial growth was evaluated by two-way ANOVA (concentration × time). IC_50_ values for MCF-7 cell viability were calculated using four-parameter nonlinear regression. Significance was defined as *p* < 0.05. All analyses were performed using GraphPad Prism (GraphPad Software, San Diego, CA, USA).

## 5. Conclusions

This study presents the first integrated, genome-resolved characterization of *Bacillus licheniformis* BLN313 as a biogenic selenium nanoparticle (SeNP) producer, linking its confirmed phenotype to the genetic determinants of selenium metabolism. Substrate optimization established 500 µg/mL of sodium selenite as the optimal concentration, at which the strain attained a high selenite-to-Se^0^ conversion efficiency of 88.81% while preserving robust growth, indicating efficient reduction within a defined working range. Comprehensive physicochemical characterization confirmed that the purified nanoparticles were spherical, predominantly amorphous with a partial trigonal Se^0^ crystalline fraction, colloidally stable, and stabilized by a proteinaceous and peptidoglycan-derived capping layer, consistent with an enzymatically mediated reduction and biomolecular stabilization mechanism. The SeNPs exhibited appreciable antibacterial activity and concentration-dependent cytotoxicity toward MCF-7 breast cancer cells while sparing normal MCF-10A cells at same concentration, supporting their functional potential for biomedical and agricultural applications. Whole-genome sequencing unambiguously reclassified the strain from *Bacillus subtilis* to *Bacillus licheniformis* on the basis of concordant ANI, dDDH, and phylogenomic evidence. Comparative genomic analysis further resolved a coordinated repertoire of thioredoxin-dependent reductases, sulfur-metabolism and oxidative-stress genes, methyltransferases, and SulP and Pit oxyanion transporters that together represent a plausible candidate pathway for selenium uptake, intracellular reduction, and redox homeostasis pending functional validation. By coupling phenotypic optimization with genome-level interpretation, this work advances the understanding of microbial selenium metabolism and establishes BLN313 as a promising platform for scalable, eco-friendly SeNP production, providing a foundation for future transcriptomic, proteomic, and metabolic engineering studies.

## Figures and Tables

**Table 1:** Antibacterial efficacy of biogenic selenium nanoparticles against Gram-positive and Gram-negative bacteria.

Organism	Treatment	ZOI (mm, Mean ± SD)	MIC (µg/mL)	MBC (µg/mL)	MBC/MIC
*M. luteus*	SeNPs	13.23 ± 0.15	62.5	125	2
	Ciprofloxacin	31.17 ± 0.09	NT	NT	—
	Sodium selenite	NI	—	—	—
	Sterile water	NI	—	—	—
*K. pneumoniae*	SeNPs	9.23 ± 0.15	250	500	2
	Ciprofloxacin	30.73 ± 0.18	NT	NT	—
	Sodium selenite	NI	—		—
	Sterile water	NI	—	—	—

Antimicrobial activity of BLN313-SeNPs against *M. luteus* and *K. pneumoniae*. ZOI values are mean ± SD (*n* = 3). MIC and MBC were determined by broth microdilution. NT: not tested; NI: no inhibition. Statistical differences were evaluated by two-way ANOVA with Tukey’s post hoc test (*p* < 0.05). Ciprofloxacin was included as a positive control for disk diffusion only. Each ciprofloxacin disc was prepared by adding 5 µL of a 1 mg/mL (1000 µg/mL) ciprofloxacin stock solution to a sterile 6 mm blank disc, giving a final content of 5 µg/disc.

**Table 2:** Summary of genomic features for strain BLN313.

Genomic Feature	Value
Genome size	4.1 Mb
Genome coverage	275 X
Q20	98.89%
Q30	96.66%
N50	10,103 bp
G + C content	46.3%
Number of plasmids	None
Genome contamination	None
Number of subsystems	463
Number of protein coding sequences	4373
Number of RNAs	105

**Table 3:** Statistics of non-coding RNA predictions in the genome of BLN313.

Type	Copy Number	Avg. Length (bp)	Total Length (bp)	Percent of Genome (%)
5S rRNA	8	111	888	0.0216
16S rRNA	8	1548	12,385	0.3010
23S rRNA	8	2927	23,416	0.5691
tRNA	82	77	6338	0.1540
ncRNA	101	148	14,969	0.3638

**Table 4:** Tandem repeat statistics in the genome of BLN313.

Total Tandem Repeats	Microsatellite DNA	Minisatellite DNA	Satellite DNA
129	0	112	2

**Table 5:** Statistics of scattered repeats in the genome of BLN313.

SINEs	LINEs	LTR	DNA Elements	Unclassified	Small RNA	Satellites	Simple Repeats	Low Complexity
20	45	123	76	9	0	2	0	0

SINEs: short interspersed nuclear elements; LINEs: long interspersed nuclear elements; LTR: long terminal repeats; DNA elements: DNA transposons; unclassified: other unclassified repeats; small RNA: small non-coding RNAs; satellites: satellite sequences; simple repeats: short tandem repeats; low complexity: low-complexity sequences.

**Table 6:** Genome comparisons of strain BLN313 with closely related type strains.

Strain (Accession No.)	16S rRNA Gene Identity (%)	dDDH (%)	ANI (%)
*Bacillus licheniformis* ATCC 14580 ^T^ (AE017333)	99.80	97.80	99.69
*Bacillus haynesii* NRRL B-41327 ^T^ (MRBL01000076)	99.73	64.20	95.62
*Bacillus sonorensis* NBRC 101234 ^T^ (AYTN01000016)	99.59	24.60	81.72
*Bacillus paralicheniformis* KJ-16 ^T^ (KY694465)	99.59	58.30	94.74
*Bacillus glycinifermentans* GO-13 ^T^ (LECW01000063)	99.39	23.60	80.89
*Bacillus swezeyi* NRRL B-41294 ^T^ (MRBK01000096)	99.05	26.40	83.18

ANI, average nucleotide identity; dDDH, digital DNA–DNA hybridization; 16S rRNA gene similarity ≥ 98.7%.

**Table 7:** Comparative analysis of key genes associated with selenium nanoparticle (SeNP) synthesis in strain BLN313 and related species.

Microorganism	Genome Size (Mb)	Key Genes Associated with Biogenic Selenium Nanoparticle (SeNP) Synthesis									
		*trxB*	*metE*	*yitJ*	*mmuM*	*ypjH*	*ypjG*	*yojG*	*yjcL*	*mccA*	Catalase Genes
BSN313 (this study)	4.11	*trxB* (6)	*metE* (2)	*yitJ* (1)	*mmuM* (1)	-	*ypjG* (1)	*yojG* (1)	*yjcL* (1)	*mccA* (1)	Catalase Genes (4)
*Bacillus licheniformis* ATCC 14580 ^T^		*trxB* (5)	*metE* (1)	*yitJ* (1)	*mmuM* (1)	-	*ypjG* (1)	*yojG* (1)	*yjcL* (1)	*mccA* (1)	Catalase Genes (5)
*Bacillus subtilis* DSM 10 ^T^	4.29	*trxB* (4)	*metE* (1)	*yitJ* (1)	*mmuM* (1)	-	*ypjG* (1)	*yojG* (1)	*yjcL* (1)	*mccA* (1)	Catalase Genes (5)
*Bacillus mycoides* ATCC 6462 ^T^	5.63	*trxB* (5)	*metE* (1)	*yitJ* (1)	*mmuM* (1)	-	*ypjG* (1)	*yojG* (2)	*yjcL* (1)	*mccA* (1)	Catalase Genes (3)
*Lactobacillus casei* DSM 20011 ^T^	2.95	*trxB* (2)	*metE* (1)	*yitJ* (1)	-	-	-	-	-	*mccA* (1)	Catalase Genes (1)
*Lactobacillus acidophilus* ATCC 4356 ^T^	1.97	*trxB* (3)	-	-	*mmuM* (1)	-	-	-	-	*mccA* (1)	-
*Escherichia coli* ATCC 25922 ^T^	5.11	*trxB* (1)	*metE* (1)	*yitJ* (1)	-	-	-	-	*yjcL* (1)	-	Catalase Genes (2)
*Bifidobacterium animalis* BB-12	1.94	*trxB* (1)	*metE* (1)	*yitJ* (1)	-	-	-	-	*yjcL* (1)	*mccA* (1)	-
*Enterobacter cloacae* DSM 30054 ^T^	5.60	*trxB* (1)	*metE* (2)	*yitJ* (1)	*mmuM* (1)	-	-	-	*yjcL* (1)	*mccA* (1)	Catalase Genes (2)
*Enterococcus faecalis* DSM 20478 ^T^	2.86	*trxB* (2)	-	-	-	-	-	-	*yjcL* (1)	*mccA* (1)	-
*Staphylococcus carnosus* FDAARGOS-883	2.57	*trxB* (3)	*metE* (1)	*yitJ* (1)	-	-	-	*yojG* (1)	-	*mccA* (1)	Catalase Genes (2)
*Pseudomonas aeruginosa* NBRC 12689 ^T^	6.31	*trxB* (5)	*metE* (1)	*yitJ* (1)	-	-	-	-	*yjcL* (1)	*mccA* (2)	Catalase Genes (4)
*Zoogloea ramigera* NBRC 15342 ^T^	4.60	*trxB* (2)	-	*yitJ* (1)	-	-	-	-	-	-	Catalase Genes (2)
*Klebsiella pneumoniae* HS11286	5.68	*trxB* (1)	*metE* (2)	*yitJ* (1)	*mmuM* (1)	-	-	-	*yjcL* (1)	*mccA* (2)	Catalase Genes (2)
*Streptococcus thermophilus* ATCC 19258 ^T^	2.10	*trxB* (2)	*metE* (6)	*yitJ* (1)	*mmuM* (1)	-	-	-	*yjcL* (1)	-	-
*Stenotrophomonas maltophilia* NCTC 10257 ^T^	5.00	*trxB* (2)	*metE* (1)	*yitJ* (2)	-	-	-	-	*yjcL* (1)	*mccA* (1)	Catalase Genes (3)

*trxB*, thioredoxin reductase; *metE*, 5-methyltetrahydropteroyltriglutamate homocysteine methyltransferase; *yitJ*, 5,10-methylenetetrahydrofolate reductase; *mmuM*, homocysteine S-methyltransferase; *ypjH*, BshA glycosyltransferase; *ypjG*, bacillithiol biosynthesis deacetylase BshB1/N-acetylglucosamin-malate deacetylase BshB; *yojG*, bacillithiol biosynthesis deacetylase BshB2; *mccA*, cystathionine beta-synthase; *yjcL*, cystathionine gamma-synthase. ^T^, type strain.

**Table 8:** Genomic comparisons of potent transporters involved in selenium oxyanion uptake in strain BLN313 and related species.

Microorganism	Transporters Encoded by Different Genes Involved in Selenium Oxyanion Transport in Biogenic SeNP-Synthesizing Bacteria									
	CysZ-Type	CysA	CysW	CysT	CysP/Sbp	SulP	PiT	ModA	ModB	ModC
BSN313 (this study)	-	-	-	-	-	SulP	PiT	ModA	ModB	-
*Bacillus licheniformis* ATCC 14580 ^T^	-	-	-	-	-	-	-	ModA	ModB	-
*Bacillus subtilis* DSM 10 ^T^	-	-	-	-	-	SulP	-	ModA	ModB	-
*Bacillus mycoides* ATCC 6462 ^T^	-	CysA	CysW	CysT	Sbp	-	-	ModA	ModB	-
*Lactobacillus casei* DSM 20011 ^T^	-	-	-	-	-	-	-	-	-	-
*Lactobacillus acidophilus* ATCC 4356 ^T^	-	-	-	-	-	-	-	-	-	-
*Escherichia coli* ATCC 25922 ^T^	CysZ	CysA	CysW	CysT	CysP	-	-	ModA	ModB	ModC
*Bifidobacterium animalis* BB-12	-	-	-	-	-	-	-	-	-	-
*Enterobacter cloacae* DSM 30054 ^T^	CysZ	CysA	CysW	CysT	CysP	SulP	PiT	ModA	ModB	-
*Enterococcus faecalis* DSM 20478 ^T^	-	-	-	-	-	-	-	ModA	ModB	ModC
*Staphylococcus carnosus* FDAARGOS-883	-	-	-	-	-	-	-	ModA	ModB	ModC
*Pseudomonas aeruginosa* NBRC 12689 ^T^	CysZ	CysA	CysW	CysT	CysP	-	-	ModA	ModB	ModC
*Zoogloea ramigera* NBRC 15342 ^T^	-	CysA	CysW	CysT	CysP	SulP	-	ModA	ModB	ModC
*Klebsiella pneumoniae* HS11286	CysZ	CysA	CysW	CysT	CysP	SulP	PiT	ModA	ModB	ModC
*Streptococcus thermophilus* ATCC 19258 ^T^	-	-	-	-	-	-	-	-	-	-
*Stenotrophomonas maltophilia* NCTC 10257 ^T^	-	-	-	-	-	-	-	-	-	-

Sulfate transporter; CysA, sulfate and thiosulfate import ATP-binding protein; CysW, sulfate transport system permease protein; CysT, sulfate transport system permease protein; CysP/Sbp, sulfate and thiosulfate binding protein; SulP, sulfate permease; PiT, sodium-dependent phosphate transporter; ChrA, chromate transport protein; ModE, molybdate-binding domain of ModE; ModABC, molybdate uptake molybdenum transport ATP-binding protein ModC transporters MolT; ModA, molybdenum ABC transporter, periplasmic molybdenum-binding protein; ModB, molybdenum transport system permease protein; ModC, molybdenum transport ATP-binding protein. ^T^, type strain.

## Data Availability

The whole-genome shotgun project of *Bacillus licheniformis* BLN313 has been archived in the DDBJ/ENA/GenBank databases under the accession number JBVVWV000000000. The version discussed in this study is JBVVWV010000000. The associated Bio Project and BioSample accession numbers are PRJNA1292399 and SAMN49999719, respectively.

## Supplementary Materials

The following supporting information can be downloaded at: https://www.mdpi.com/article/10.3390/ijms27177792/s1.

## Abbreviations

The following abbreviations are used in this manuscript:

ANI	average nucleotide identity
dDDH	digital DNA–DNA hybridization
DMEM	Dulbecco’s Modified Eagle’s Medium
DMSO	dimethyl sulfoxide
EDS	energy-dispersive X-ray spectroscopy
FBS	fetal bovine serum
FTIR	Fourier-transform infrared spectroscopy
GC	guanine + cytosine
IC_50_	half-maximal inhibitory concentration
LC-AFS	liquid chromatography coupled with atomic fluorescence spectrometry
LB	Luria–Bertani
MBC	minimum bactericidal concentration
MIC	minimum inhibitory concentration
MTT	3-(4,5-dimethylthiazol-2-yl)-2,5-diphenyltetrazolium bromide
ncRNA	non-coding RNA
NEAAs	non-essential amino acids
NPs	nanoparticles
OGRI	overall genome-related index
ORFs	open reading frames
PIT	sodium-dependent phosphate transporter
RAST	Rapid Annotation using Subsystem Technology
ROS	reactive oxygen species
rRNA	ribosomal RNA
Se	selenium
SEM	scanning electron microscopy
SeNPs	selenium nanoparticles
SDS	sodium dodecyl sulfate
SulP	sulfate permease
TEM	transmission electron microscopy
tRNA	transfer RNA
TYGS	Type (Strain) Genome Server
XRD	X-ray diffraction
ZOI	zone of inhibition
