# Supplementary material for: Integrated Optimization, Genomic Characterization, and Functional Evaluation of Biogenic Selenium Nanoparticles from Bacillus licheniformis BLN313: Antibacterial and Anticancer Potential
Source: Int J Mol Sci. 2026 Aug 31;27(17):7792. doi: 10.3390/ijms27177792 (PMC13566226; doi:10.3390/ijms27177792)
Supplement: Supplementary file 1 [file ijms-27-07792-s001.zip › Supplementary figures.pdf]

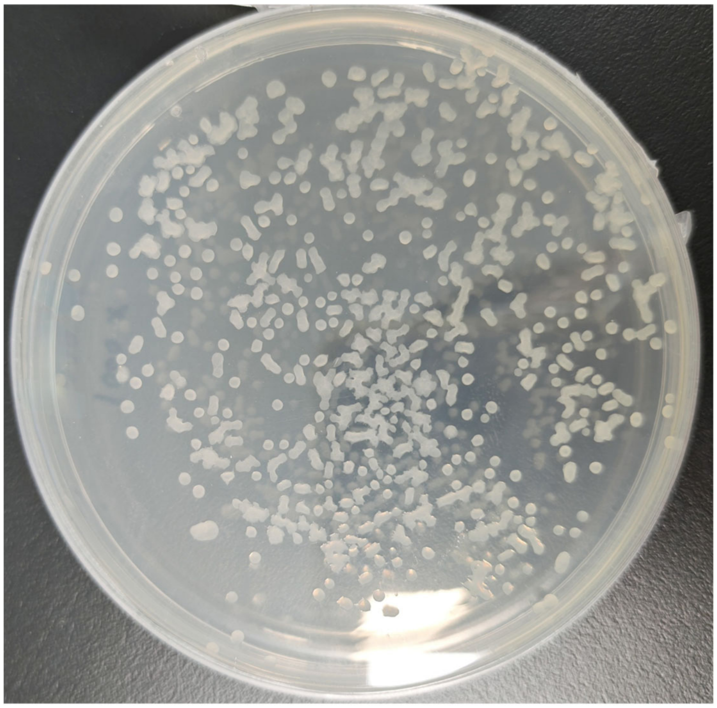

Figure S1. Growth of *Bacillus licheniformis* BLN313 on LB agar plates

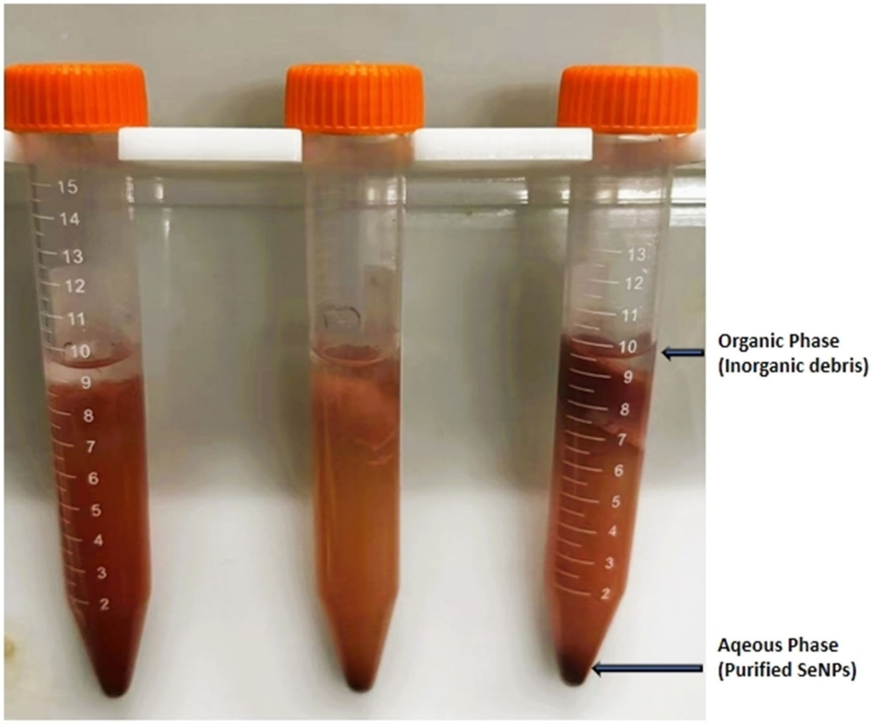

Figure S2. Extraction and purification of selenium nanoparticles (SeNPs) by a two-phase separation method, resulting in separation into an inorganic debris phase and an aqueous phase containing purified, reddish-colored SeNPs.

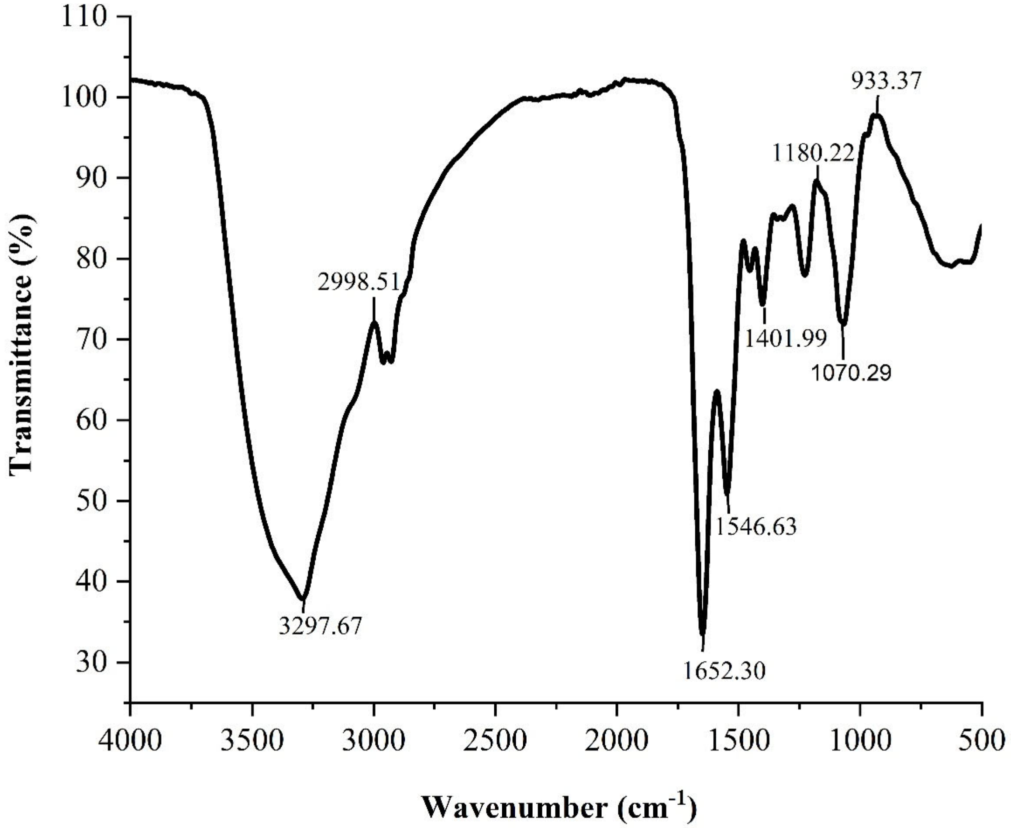

Figure S3. FTIR spectrum of untreated *Bacillus licheniformis* BLN313 control sample.

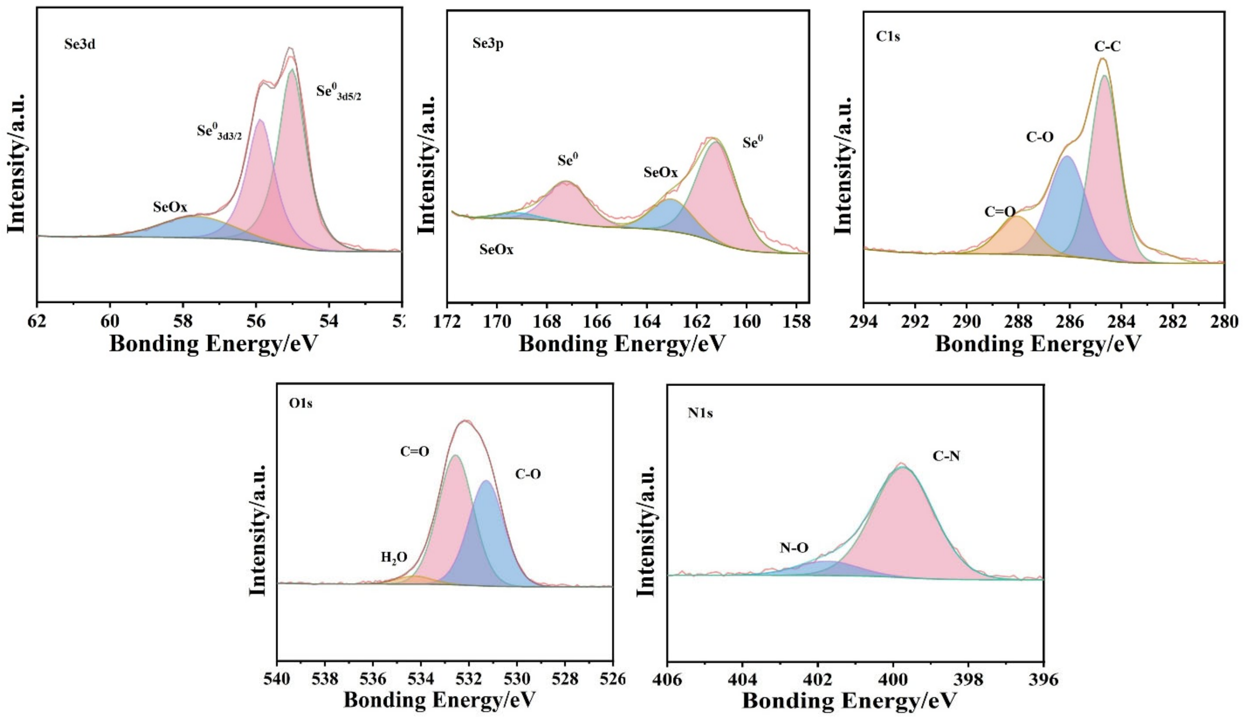

Figure S4. High-resolution deconvoluted XPS spectra of BLN313-SeNPs, including Se 3d, Se 3p, C 1s, O 1s, and N 1s regions, showing Se^{0}/SeOx species and biomolecule-derived C-, O-, and N-containing surface functionalities.

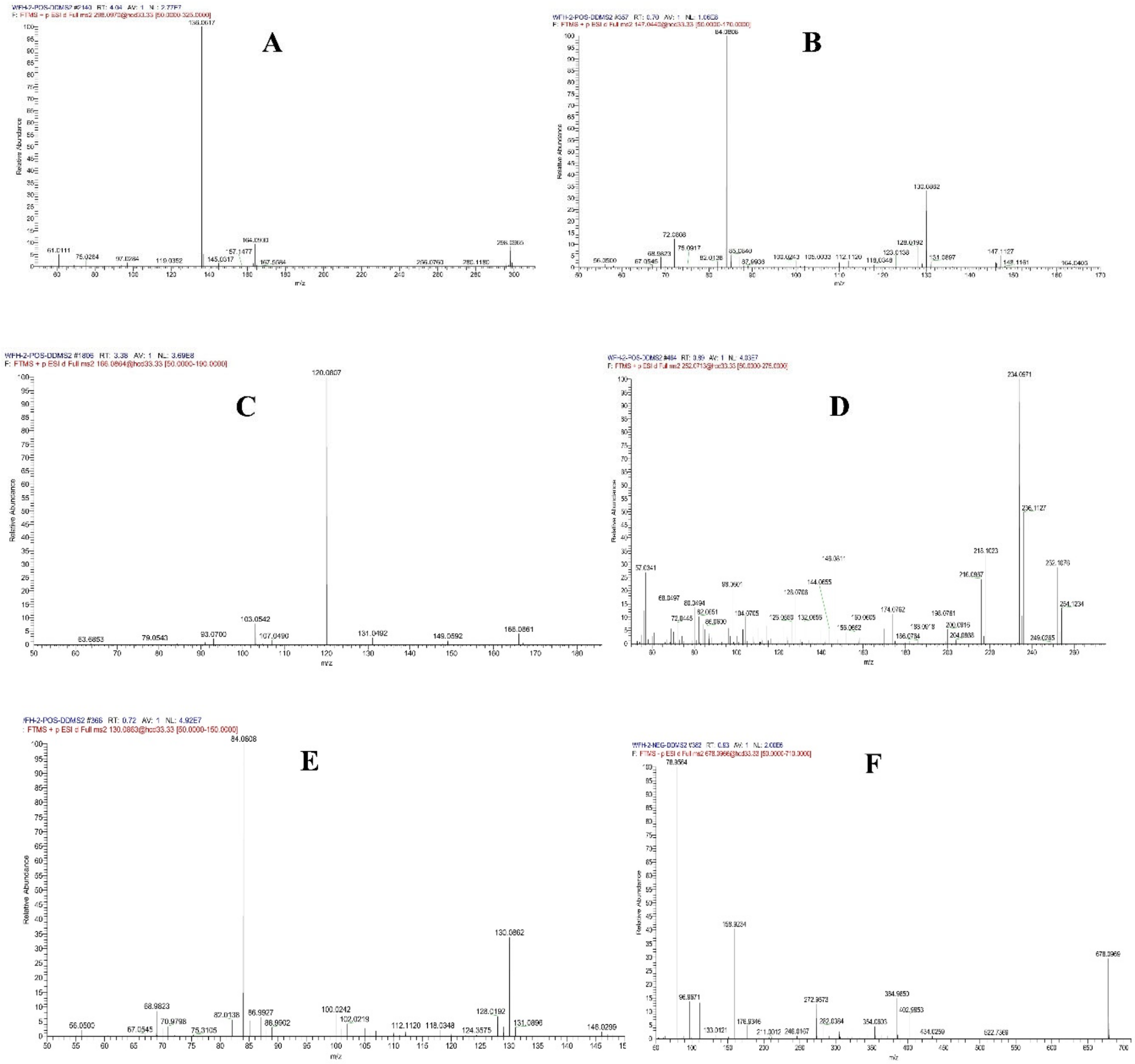

Figure S5. Representative mass spectra of the identified metabolites: (A) 5′-methylthioadenosine, (B) L-lysine, (C) L-phenylalanine, (D) muramic acid, (E) pipecolic acid, and (F) UDP-N-acetylmuramate. The spectra show the characteristic mass-to-charge (*m/z*) signals corresponding to each detected metabolite.

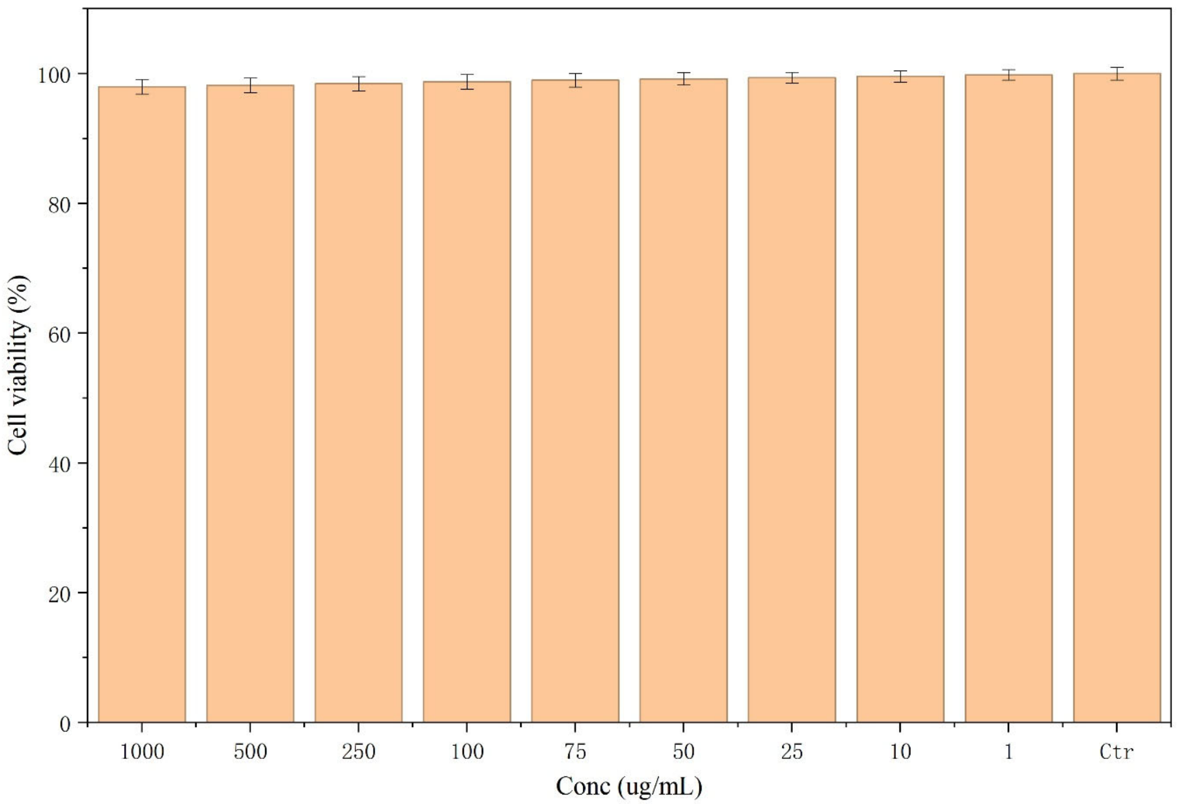

Figure S6. Cell viability of MCF-10A normal mammary epithelial cells following BLN313-SeNP exposure, showing maintained viability above 97% across the tested concentration range of 1–1000 µg/mL compared with the untreated control.
