# Supplementary material for: Integrated Optimization, Genomic Characterization, and Functional Evaluation of Biogenic Selenium Nanoparticles from Bacillus licheniformis BLN313: Antibacterial and Anticancer Potential
Source: Int J Mol Sci. 2026 Aug 31;27(17):7792. doi: 10.3390/ijms27177792 (PMC13566226; doi:10.3390/ijms27177792)
Supplement: Supplementary file 1 [file ijms-27-07792-s001.zip › Supplementary tables 3 and 4.pdf]

Supplementary Table S3. Complete list of genes and transporters identified in strain BLN313 and related species through RAST genome annotation

Species Name	Category	Subcategory	Subsystem	Role	Features
**BLN313**	Sulfur Metabolism	Sulfur Metabolism - no subcategory	Thioredoxin-disulfide reductase	Thioredoxin reductase (EC 1.8.1.9), ***trxB***	fig|1402.1084.peg.760, fig|1402.1084.peg.2419, fig|1402.1084.peg.2827, fig|1402.1084.peg.2828, fig|1402.1084.peg.3415, fig|1402.1084.peg.3713
	Nucleosides and Nucleotides	Sulfur Metabolism - no subcategory	Thioredoxin-disulfide reductase	Thioredoxin reductase (EC 1.8.1.9), ***trxB***	fig|1402.1084.peg.760, fig|1402.1084.peg.2419, fig|1402.1084.peg.2827, fig|1402.1084.peg.2828, fig|1402.1084.peg.3415, fig|1402.1084.peg.3713
	Amino Acids and Derivatives	Lysine, threonine, methionine, and cysteine	Methionine Biosynthesis	5-methyltetrahydrofolate--homocysteine methyltransferase (EC 2.1.1.13), ***metE***	fig|1402.1084.peg.1209
	Amino Acids and Derivatives	Lysine, threonine, methionine, and cysteine	Methionine Biosynthesis	5-methyltetrahydropteroyltriglutamate--homocysteine methyltransferase (EC 2.1.1.14), ***metE***	fig|1402.1084.peg.1455
	Cofactors, Vitamins, Prosthetic Groups, Pigments	Folate and pterines	5-FCL-like protein	5,10-methylenetetrahydrofolate reductase (EC 1.5.1.20), ***yitJ***	fig|1402.1084.peg.1210
	Amino Acids and Derivatives	Lysine, threonine, methionine, and cysteine	Methionine Biosynthesis	5,10-methylenetetrahydrofolate reductase (EC 1.5.1.20), ***yitJ***	fig|1402.1084.peg.1210
	Carbohydrates	One-carbon Metabolism	Serine-glyoxylate cycle	5,10-methylenetetrahydrofolate reductase (EC 1.5.1.20), ***yitJ***	fig|1402.1084.peg.1210
	Carbohydrates	One-carbon Metabolism	One-carbon metabolism by tetrahydropterines	5,10-methylenetetrahydrofolate reductase (EC 1.5.1.20), ***yitJ***	fig|1402.1084.peg.1210
	Amino Acids and Derivatives	Lysine, threonine, methionine, and cysteine	S-methylmethionine	Homocysteine S-methyltransferase (EC 2.1.1.10), ***MmuM***	fig|1402.1084.peg.242
	Amino Acids and Derivatives	Lysine, threonine, methionine, and cysteine	Methionine Biosynthesis	Homocysteine S-methyltransferase (EC 2.1.1.10), ***MmuM***	fig|1402.1084.peg.242
	Amino Acids and Derivatives	Alanine, serine, and glycine	Glycine and Serine Utilization	Cystathionine beta-synthase (EC 4.2.1.22), ***mccA***	fig|1402.1084.peg.2878
	Amino Acids and Derivatives	Lysine, threonine, methionine, and cysteine	Methionine Degradation	Cystathionine beta-synthase (EC 4.2.1.22), ***mccA***	fig|1402.1084.peg.2878
	Amino Acids and Derivatives	Lysine, threonine, methionine, and cysteine	Methionine Biosynthesis	Cystathionine beta-synthase (EC 4.2.1.22), **mccA**	fig|1402.1084.peg.2878
	Sulfur metabolism	Organic sulfur assimilation	L-Cystine Uptake and Metabolism	Cystathionine gamma-synthase (EC 2.5.1.48), ***yjcL***	fig|1402.1084.peg.1316
	Amino Acids and Derivatives	Lysine, threonine, methionine, and cysteine	Methionine Biosynthesis	Cystathionine gamma-synthase (EC 2.5.1.48), ***yjcL***	fig|1402.1084.peg.1316
				ypjG (KEGG Id: K01463)	
				yojG (KEGG Id: K22135)	
	Stress Response	Oxidative stress	Protection from Reactive Oxygen Species	Catalase (EC 1.11.1.6)	fig|1402.1084.peg.1963, fig|1402.1084.peg.4118, fig|1402.1084.peg.4120, fig|1402.1084.peg.4201
	Stress Response	Oxidative stress	Oxidative stress	Catalase (EC 1.11.1.6)	fig|1402.1084.peg.1963, fig|1402.1084.peg.4118, fig|1402.1084.peg.4120, fig|1402.1084.peg.4201
	-	-	-	Sulfate permease, ***SulP***	fig|1402.1084.peg.1714 fig|1402.1084.peg.2109 fig|1402.1084.peg.4350 fig|1402.1084.peg.3699
	Membrane Transport	Uni- Sym- and Antiporters	NhaA, NhaD and Sodium-dependent phosphate transporters	Sodium-dependent phosphate transporter, ***PiT***	fig|1402.1084.peg.223, fig|1402.1084.peg.2732
	Cofactors, Vitamins, Prosthetic Groups, Pigments	Folate and pterines	Molybdenum cofactor biosynthesis	Molybdenum ABC transporter, periplasmic molybdenum-binding protein ModA (TC 3.A.1.8.1), ***modA***	fig|1402.1084.peg.1171

	Cofactors, Vitamins, Prosthetic Groups, Pigments	Folate and pterines	Molybdenum cofactor biosynthesis	Molybdenum transport system permease protein ModB (TC 3.A.1.8.1), ***modB***	fig|1402.1084.peg.1172
***Bacillus licheniformis* ATCC 14580**					
	Sulfur Metabolism	Sulfur Metabolism - no subcategory	Thioredoxin-disulfide reductase	Thioredoxin reductase (EC 1.8.1.9), ***trxB***	fig|1402.1080.peg.1287, fig|1402.1080.peg.1644, fig|1402.1080.peg.2226, fig|1402.1080.peg.2625, fig|1402.1080.peg.3000
	Nucleosides and Nucleotides	Pyrimidines	pyrimidine conversions	Thioredoxin reductase (EC 1.8.1.9, ***trxB***	fig|1402.1080.peg.1287, fig|1402.1080.peg.1644, fig|1402.1080.peg.2226, fig|1402.1080.peg.2625, fig|1402.1080.peg.3000
	Amino Acids and Derivatives	Lysine, threonine, methionine, and cysteine	Methionine Biosynthesis	5-methyltetrahydrofolate--homocysteine methyltransferase (EC 2.1.1.13), ***metE***	fig|1402.1080.peg.3938
	Amino Acids and Derivatives	Lysine, threonine, methionine, and cysteine	Methionine Biosynthesis	5,10-methylenetetrahydrofolate reductase (EC 1.5.1.20), ***yitJ***	fig|1402.1080.peg.3937
	Amino Acids and Derivatives	Lysine, threonine, methionine, and cysteine	Methionine Biosynthesis	Homocysteine S-methyltransferase (EC 2.1.1.10), ***MmuM***	fig|1402.1080.peg.361
	Amino Acids and Derivatives	Lysine, threonine, methionine, and cysteine	Methionine Biosynthesis	Cystathionine beta-synthase (EC 4.2.1.22), ***mccA***	fig|1402.1080.peg.2175
	Amino Acids and Derivatives	Alanine, serine, and glycine	Glycine and Serine Utilization	Cystathionine beta-synthase (EC 4.2.1.22), ***mccA***	fig|1402.1080.peg.2175
	Amino Acids and Derivatives	Lysine, threonine, methionine, and cysteine	Methionine Biosynthesis	Cystathionine gamma-synthase (EC 2.5.1.48), ***yjcL***	fig|1402.1080.peg.3833
	Sulfur Metabolism	Organic sulfur assimilation	L-Cystine Uptake and Metabolism	Cystathionine gamma-synthase (EC 2.5.1.48), ***yjcL***	fig|1402.1080.peg.3833
				ypjG - bacillithiol biosynthesis deacetylase BshB1 [GenBank: WQG64240.1]	
				yojG - bacillithiol biosynthesis deacetylase BshB2 [GenBank: WQG60654.1]	
	Stress Response	Oxidative stress	Oxidative stress	Catalase (EC 1.11.1.6)	fig|1402.1080.peg.790, fig|1402.1080.peg.791, fig|1402.1080.peg.878, fig|1402.1080.peg.880, fig|1402.1080.peg.3081
	Cofactors, Vitamins, Prosthetic Groups, Pigments	Folate and pterines	Molybdenum cofactor biosynthesis	Molybdenum ABC transporter, periplasmic molybdenum-binding protein ModA (TC 3.A.1.8.1), ***modA***	fig|1402.1080.peg.3978
	Cofactors, Vitamins, Prosthetic Groups, Pigments	Folate and pterines	Molybdenum cofactor biosynthesis	Molybdenum transport system permease protein ModB (TC 3.A.1.8.1), ***modB***	fig|1402.1080.peg.3977
***Bacillus subtilis* DSM 10**					
	Nucleosides and Nucleotides	Pyrimidines	pyrimidine conversions	Thioredoxin reductase (EC 1.8.1.9), ***trxB***	fig|1423.3804.peg.1911, fig|1423.3804.peg.2894, fig|1423.3804.peg.3187, fig|1423.3804.peg.4209
	Sulfur Metabolism	Sulfur Metabolism - no subcategory	Thioredoxin-disulfide reductase	Thioredoxin reductase (EC 1.8.1.9), ***trxB***	fig|1423.3804.peg.1911, fig|1423.3804.peg.2894, fig|1423.3804.peg.3187, fig|1423.3804.peg.4209
	Amino Acids and Derivatives	Lysine, threonine, methionine, and cysteine	Methionine Biosynthesis	5-methyltetrahydropteroyltriglutamate--homocysteine methyltransferase (EC 2.1.1.14), ***metE***	fig|1423.3804.peg.885
	Carbohydrates	One-carbon Metabolism	One-carbon metabolism by tetrahydropterines	5,10-methylenetetrahydrofolate reductase (EC 1.5.1.20), ***yitJ***	fig|1423.3804.peg.636
	Carbohydrates	One-carbon Metabolism	Serine-glyoxylate cycle	5,10-methylenetetrahydrofolate reductase (EC 1.5.1.20), ***yitJ***	fig|1423.3804.peg.636
	Amino Acids and Derivatives	Lysine, threonine, methionine, and cysteine	Methionine Biosynthesis	5,10-methylenetetrahydrofolate reductase (EC 1.5.1.20), ***yitJ***	fig|1423.3804.peg.636
	Cofactors, Vitamins, Prosthetic Groups, Pigments	Folate and pterines	5-FCL-like protein	5,10-methylenetetrahydrofolate reductase (EC 1.5.1.20), ***yitJ***	fig|1423.3804.peg.636

				MmuM<br>**-**<br>Homocysteine S-methyltransferase GenBank: WFA12205.1	
	Amino Acids and Derivatives	Lysine, threonine, methionine, and cysteine	Methionine Degradation	Cystathionine beta-synthase (EC 4.2.1.22), ***mccA***	fig|1423.3804.peg.2371
	Amino Acids and Derivatives	Alanine, serine, and glycine	Glycine and Serine Utilization	Cystathionine beta-synthase (EC 4.2.1.22), ***mccA***	fig|1423.3804.peg.2371
	Sulfur Metabolism	Organic sulfur assimilation	L-Cystine Uptake and Metabolism	Cystathionine gamma-synthase (EC 2.5.1.48), ***yjcL***	fig|1423.3804.peg.733,
	Amino Acids and Derivatives	Lysine, threonine, methionine, and cysteine	Methionine Biosynthesis	Cystathionine gamma-synthase (EC 2.5.1.48), ***yjcL***	fig|1423.3804.peg.733,
				ypjG<br>–<br>bacillithiol biosynthesis deacetylase BshB1 [GenBank: WFA14200.1	
				yojG<br>–<br>bacillithiol biosynthesis deacetylase BshB2 [GenBank: WFA13896.1]	
	Stress Response	Oxidative stress	Protection from Reactive Oxygen Species	Catalase (EC 1.11.1.6)	fig|1423.3804.peg.407, fig|1423.3804.peg.805, fig|1423.3804.peg.3604, fig|1423.3804.peg.3649, fig|1423.3804.peg.4340
	Sulfur Metabolism	Inorganic sulfur assimilation	Inorganic Sulfur Assimilation	Sulfate permease, Pit-type, ***SulP***	fig|1423.3804.peg.1153
	Cofactors, Vitamins, Prosthetic Groups, Pigments	Folate and pterines	Molybdenum cofactor biosynthesis	Molybdenum ABC transporter, periplasmic molybdenum-binding protein ***modA*** (TC 3.A.1.8.1)	fig|1423.3804.peg.3040
	Cofactors, Vitamins, Prosthetic Groups, Pigments	Folate and pterines	Molybdenum cofactor biosynthesis	Molybdenum transport system permease protein ***modB*** (TC 3.A.1.8.1)	fig|1423.3804.peg.3041
***Bacillus mycoides* ATCC 6462**					
	Sulfur Metabolism	Sulfur Metabolism - no subcategory	Thioredoxin-disulfide reductase	Thioredoxin reductase (EC 1.8.1.9), ***trxB***	fig|1405.407.peg.530, fig|1405.407.peg.1254, fig|1405.407.peg.1454, fig|1405.407.peg.3640, fig|1405.407.peg.4833
	Nucleosides and Nucleotides	Pyrimidines	pyrimidine conversions	Thioredoxin reductase (EC 1.8.1.9), ***trxB***	fig|1405.407.peg.530, fig|1405.407.peg.1254, fig|1405.407.peg.1454, fig|1405.407.peg.3640, fig|1405.407.peg.4833
	Amino Acids and Derivatives	Lysine, threonine, methionine, and cysteine	Methionine Biosynthesis	5-methyltetrahydropteroyltriglutamate--homocysteine methyltransferase (EC 2.1.1.14), ***metE***	fig|1405.407.peg.2375
	Amino Acids and Derivatives	Lysine, threonine, methionine, and cysteine	Methionine Biosynthesis	5,10-methylenetetrahydrofolate reductase (EC 1.5.1.20), ***yitJ***	fig|1405.407.peg.211
				MmuM<br>**-**<br>homocysteine S-methyltransferase family protein [Bacillus mycoides] GenBank: AJH17988.1	
	Amino Acids and Derivatives	Lysine, threonine, methionine, and cysteine	Methionine Degradation	Cystathionine beta-synthase (EC 4.2.1.22), ***mccA***	fig|1405.407.peg.2003
	Amino Acids and Derivatives	Lysine, threonine, methionine, and cysteine	Methionine Biosynthesis	Cystathionine beta-synthase (EC 4.2.1.22), ***mccA***	fig|1405.407.peg.2003
	Amino Acids and Derivatives	Lysine, threonine, methionine, and cysteine	Methionine Biosynthesis	Cystathionine gamma-synthase (EC 2.5.1.48), ***yjcL***	fig|1405.407.peg.2114
	-	-	-	***ypjG***<br>-<br>bacillithiol biosynthesis deacetylase BshB1 [GenBank: AJH21942.1]	-
	-	-	-	***yojG***<br>-<br>bacillithiol biosynthesis deacetylase BshB2 [GenBank: AJH19854.1]	-
	Stress Response	Oxidative stress	Oxidative stress	Catalase (EC 1.11.1.6)	fig|1405.407.peg.1607, fig|1405.407.peg.3377,

					fig|1405.407.peg.5183
	Stress Response	Detoxification	Uptake of selenate and selenite	Sulfate and thiosulfate import ATP-binding protein **CysA** (EC 3.6.3.25)	fig|1405.407.peg.5232,
	Sulfur Metabolism	Inorganic sulfur assimilation	Inorganic Sulfur Assimilation	Sulfate transport system permease protein **CysW**	fig|1405.407.peg.5234
	Sulfur Metabolism	Inorganic sulfur assimilation	Inorganic Sulfur Assimilation	Sulfate transport system permease protein **CysT**	fig|1405.407.peg.5235
	Sulfur Metabolism	Inorganic sulfur assimilation	Inorganic Sulfur Assimilation	Sulfate-binding protein **Sbp**	fig|1405.407.peg.5236
	Cofactors, Vitamins, Prosthetic Groups, Pigments	Folate and pterines	Molybdenum cofactor biosynthesis	Molybdenum ABC transporter, periplasmic molybdenum-binding protein **ModA** (TC 3.A.1.8.1)	fig|1405.407.peg.675
	Cofactors, Vitamins, Prosthetic Groups, Pigments	Folate and pterines	Molybdenum cofactor biosynthesis	Molybdenum transport system permease protein **ModB** (TC 3.A.1.8.1)	fig|1405.407.peg.676
***Lactobacillus casei* DSM 20011**					
	Sulfur Metabolism	Sulfur Metabolism - no subcategory	Thioredoxin-disulfide reductase	Thioredoxin reductase (EC 1.8.1.9), ***trxB***	fig|655183.24.peg.639, fig|655183.24.peg.851
	Nucleosides and Nucleotides	Pyrimidines	pyrimidine conversions	Thioredoxin reductase (EC 1.8.1.9), ***trxB***	fig|655183.24.peg.639, fig|655183.24.peg.851
	Amino Acids and Derivatives	Lysine, threonine, methionine, and cysteine	Methionine Biosynthesis	5-methyltetrahydropteroyltriglutamate--homocysteine methyltransferase (EC 2.1.1.14), ***metE***	fig|655183.24.peg.425
	Amino Acids and Derivatives	Lysine, threonine, methionine, and cysteine	Methionine Biosynthesis	5,10-methylenetetrahydrofolate reductase (EC 1.5.1.20), ***yitJ***	fig|655183.24.peg.424
	Amino Acids and Derivatives	Lysine, threonine, methionine, and cysteine	Methionine Degradation	Cystathionine beta-synthase (EC 4.2.1.22), ***mccA***	fig|655183.24.peg.2859
	Amino Acids and Derivatives	Lysine, threonine, methionine, and cysteine	Cysteine Biosynthesis	Cystathionine beta-synthase (EC 4.2.1.22), ***mccA***	fig|655183.24.peg.2859
	Amino Acids and Derivatives	Lysine, threonine, methionine, and cysteine	Methionine Biosynthesis	Cystathionine beta-synthase (EC 4.2.1.22), ***mccA***	fig|655183.24.peg.2859
	Stress Response	Oxidative stress	Oxidative stress	Catalase (EC 1.11.1.6)	fig|655183.24.peg.2537
***Lactobacillus acidophilus* ATCC 4356**					
	Sulfur Metabolism	Sulfur Metabolism - no subcategory	Thioredoxin-disulfide reductase	Thioredoxin reductase (EC 1.8.1.9), ***trxB***	fig|1579.961.peg.1075, fig|1579.961.peg.1302, fig|1579.961.peg.1801
	Nucleosides and Nucleotides	Pyrimidines	pyrimidine conversions	Thioredoxin reductase (EC 1.8.1.9), ***trxB***	fig|1579.961.peg.1075, fig|1579.961.peg.1302, fig|1579.961.peg.1801
	Amino Acids and Derivatives	Lysine, threonine, methionine, and cysteine	Methionine Biosynthesis	Homocysteine S-methyltransferase (EC 2.1.1.10), ***MmuM***	fig|1579.961.peg.760
	Amino Acids and Derivatives	Lysine, threonine, methionine, and cysteine	S-methylmethionine	Homocysteine S-methyltransferase (EC 2.1.1.10), ***MmuM***	fig|1579.961.peg.760
	Amino Acids and Derivatives	Lysine, threonine, methionine, and cysteine	Methionine Degradation	Cystathionine beta-synthase (EC 4.2.1.22), ***mccA***	fig|1579.961.peg.679
	Amino Acids and Derivatives	Lysine, threonine, methionine, and cysteine	Methionine Biosynthesis	Cystathionine beta-synthase (EC 4.2.1.22), ***mccA***	fig|1579.961.peg.679
	Amino Acids and Derivatives	Alanine, serine, and glycine	Glycine and Serine Utilization	Cystathionine beta-synthase (EC 4.2.1.22), ***mccA***	fig|1579.961.peg.679
***Escherichia coli* ATCC 25922**					
	Nucleosides and Nucleotides	Pyrimidines	pyrimidine conversions	Thioredoxin reductase (EC 1.8.1.9), ***trxB***	fig|1322345.831.peg.4808
	Sulfur Metabolism	Sulfur Metabolism - no subcategory	Thioredoxin-disulfide reductase	Thioredoxin reductase (EC 1.8.1.9), ***trxB***	fig|1322345.831.peg.4808
	Amino Acids and Derivatives	Lysine, threonine, methionine, and cysteine	Methionine Biosynthesis	5-methyltetrahydropteroyltriglutamate--homocysteine methyltransferase (EC 2.1.1.14), ***metE***	fig|1322345.831.peg.1083
	Amino Acids and Derivatives	Lysine, threonine, methionine, and cysteine	Methionine Biosynthesis	5,10-methylenetetrahydrofolate reductase (EC 1.5.1.20), ***yitJ***	fig|1322345.831.peg.3026
	Sulfur Metabolism	Organic sulfur assimilation	L-Cystine Uptake and Metabolism	Cystathionine gamma-synthase (EC 2.5.1.48), ***yjcL***	fig|1322345.831.peg.3019

	Amino Acids and Derivatives	Lysine, threonine, methionine, and cysteine	Methionine Biosynthesis	Cystathionine gamma-synthase (EC 2.5.1.48), ***yjcL***	fig|1322345.831.peg.3019
	Stress Response	Oxidative stress	Oxidative stress	Catalase (EC 1.11.1.6)	fig|1322345.831.peg.2231, fig|1322345.831.peg.3027
	Sulfur Metabolism	Inorganic sulfur assimilation	Inorganic Sulfur Assimilation	Sulfate transporter, **CysZ**-type	fig|1322345.831.peg.1833
	Sulfur Metabolism	Inorganic sulfur assimilation	Inorganic Sulfur Assimilation	Sulfate and thiosulfate import ATP-binding protein **CysA** (EC 3.6.3.25)	fig|1322345.831.peg.1841
	Sulfur Metabolism	Inorganic sulfur assimilation	Inorganic Sulfur Assimilation	Sulfate transport system permease protein **CysW**	fig|1322345.831.peg.1842
	ulfur Metabolism	Inorganic sulfur assimilation	Inorganic Sulfur Assimilation	Sulfate transport system permease protein **CysT**	fig|1322345.831.peg.1843
	Sulfur Metabolism	Inorganic sulfur assimilation	Inorganic Sulfur Assimilation	Sulfate and thiosulfate binding protein **CysP**	fig|1322345.831.peg.1844
	Sulfur Metabolism	Inorganic sulfur assimilation	Inorganic Sulfur Assimilation	Sulfate-binding protein **Sbp**	fig|1322345.831.peg.2997
	Cofactors, Vitamins, Prosthetic Groups, Pigments	Folate and pterines	Molybdenum cofactor biosynthesis	Molybdenum ABC transporter, periplasmic molybdenum-binding protein **ModA** (TC 3.A.1.8.1)	fig|1322345.831.peg.4680
	Cofactors, Vitamins, Prosthetic Groups, Pigments	Folate and pterines	Molybdenum cofactor biosynthesis	Molybdenum transport system permease protein **ModB** (TC 3.A.1.8.1)	fig|1322345.831.peg.4681
	Cofactors, Vitamins, Prosthetic Groups, Pigments	Folate and pterines	Molybdenum cofactor biosynthesis	Molybdenum transport ATP-binding protein **ModC** (TC 3.A.1.8.1)	fig|1322345.831.peg.4682
***Bifidobacterium animalis* BB-12**					
	Sulfur Metabolism	Sulfur Metabolism - no subcategory	Thioredoxin-disulfide reductase	Thioredoxin reductase (EC 1.8.1.9), ***trxB***	fig|552531.42.peg.1609
	Amino Acids and Derivatives	Lysine, threonine, methionine, and cysteine	Methionine Biosynthesis	5-methyltetrahydropteroyltriglutamate--homocysteine methyltransferase (EC 2.1.1.14), ***metE***	fig|552531.42.peg.88
	Amino Acids and Derivatives	Lysine, threonine, methionine, and cysteine	Methionine Biosynthesis	5,10-methylenetetrahydrofolate reductase (EC 1.5.1.20), ***yitJ***	fig|552531.42.peg.880
	Carbohydrates	One-carbon Metabolism	One-carbon metabolism by tetrahydropterines	5,10-methylenetetrahydrofolate reductase (EC 1.5.1.20), ***yitJ***	fig|552531.42.peg.880
	Amino Acids and Derivatives	Lysine, threonine, methionine, and cysteine	Methionine Biosynthesis	Cystathionine beta-synthase (EC 4.2.1.22), ***mccA***	fig|552531.42.peg.573
***Enterobacter cloacae* DSM 30054**					
	Sulfur Metabolism	Sulfur Metabolism - no subcategory	Thioredoxin-disulfide reductase	Thioredoxin reductase (EC 1.8.1.9), ***trxB***	fig|550.5374.peg.5053
	Nucleosides and Nucleotides	Pyrimidines	pyrimidine conversions	Thioredoxin reductase (EC 1.8.1.9), ***trxB***	fig|550.5374.peg.5053
	Amino Acids and Derivatives	Lysine, threonine, methionine, and cysteine	Methionine Biosynthesis	5-methyltetrahydropteroyltriglutamate--homocysteine methyltransferase (EC 2.1.1.14), ***metE***	fig|550.5374.peg.1919, fig|550.5374.peg.4403
	Amino Acids and Derivatives	Lysine, threonine, methionine, and cysteine	Methionine Biosynthesis	5,10-methylenetetrahydrofolate reductase (EC 1.5.1.20), ***yitJ***	fig|550.5374.peg.1850
	Amino Acids and Derivatives	Lysine, threonine, methionine, and cysteine	Methionine Biosynthesis	Homocysteine S-methyltransferase (EC 2.1.1.10), ***MmuM***	fig|550.5374.peg.646
	Amino Acids and Derivatives	Lysine, threonine, methionine, and cysteine	S-methylmethionine	Homocysteine S-methyltransferase (EC 2.1.1.10), ***MmuM***	fig|550.5374.peg.646
	Amino Acids and Derivatives	Lysine, threonine, methionine, and cysteine	Methionine Biosynthesis	Cystathionine beta-synthase (EC 4.2.1.22), ***mccA***	fig|550.5374.peg.4658
	Amino Acids and Derivatives	Lysine, threonine, methionine, and cysteine	Cysteine Biosynthesis	Cystathionine beta-synthase (EC 4.2.1.22), ***mccA***	fig|550.5374.peg.4658
	Amino Acids and Derivatives	Lysine, threonine, methionine, and cysteine	Methionine Degradation	Cystathionine beta-synthase (EC 4.2.1.22), ***mccA***	fig|550.5374.peg.4658
	Amino Acids and Derivatives	Lysine, threonine, methionine, and cysteine	Methionine Biosynthesis	Cystathionine gamma-synthase (EC 2.5.1.48), ***yjcL***	fig|550.5374.peg.1848
	Stress Response	Oxidative stress	Oxidative stress	Catalase (EC 1.11.1.6)	fig|550.5374.peg.1851, fig|550.5374.peg.4729
	Amino Acids and Derivatives	Lysine, threonine, methionine, and cysteine	Cysteine Biosynthesis	Sulfate transporter, **CysZ**-type	fig|550.5374.peg.3115

	Stress Response	Detoxification	Uptake of selenate and selenite	Sulfate and thiosulfate import ATP-binding protein **CysA** (EC 3.6.3.25)	fig|550.5374.peg.3108
	Amino Acids and Derivatives	Lysine, threonine, methionine, and cysteine	Cysteine Biosynthesis	Sulfate transport system permease protein **CysW**	fig|550.5374.peg.3107
	Amino Acids and Derivatives	Lysine, threonine, methionine, and cysteine	Cysteine Biosynthesis	Sulfate transport system permease protein **CysT**	fig|550.5374.peg.3106
	Amino Acids and Derivatives	Lysine, threonine, methionine, and cysteine	Cysteine Biosynthesis	Sulfate and thiosulfate binding protein **CysP**	fig|550.5374.peg.3105
	Amino Acids and Derivatives	Lysine, threonine, methionine, and cysteine	Cysteine Biosynthesis	Sulfate permease, **SulP**	fig|550.5374.peg.1254,
	Sulfur Metabolism	Inorganic sulfur assimilation	Inorganic Sulfur Assimilation	Putative sulfate permease, **SulP**	fig|550.5374.peg.3911
	Membrane Transport	Uni- Sym- and Antiporters	NhaA, NhaD and Sodium-dependent phosphate transporters	Sodium-dependent phosphate transporter, **PiT**	fig|550.5374.peg.1457
	Cofactors, Vitamins, Prosthetic Groups, Pigments	Folate and pterines	Molybdenum cofactor biosynthesis	Molybdenum ABC transporter, periplasmic molybdenum-binding protein **ModA** (TC 3.A.1.8.1)	fig|550.5374.peg.201
	Cofactors, Vitamins, Prosthetic Groups, Pigments	Folate and pterines	Molybdenum cofactor biosynthesis	Molybdenum transport system permease protein **ModB** (TC 3.A.1.8.1)	fig|550.5374.peg.200
	Cofactors, Vitamins, Prosthetic Groups, Pigments	Folate and pterines	Molybdenum cofactor biosynthesis	Molybdenum transport ATP-binding protein **ModC** (TC 3.A.1.8.1)	fig|550.5374.peg.199
***Enterococcus faecalis* DSM 20478**					
	Nucleosides and Nucleotides	Pyrimidines	pyrimidine conversions	Thioredoxin reductase (EC 1.8.1.9), ***trxB***	fig|1351.11559.peg.1149, fig|1351.11559.peg.2376
	Sulfur Metabolism	Sulfur Metabolism - no subcategory	Thioredoxin-disulfide reductase	Thioredoxin reductase (EC 1.8.1.9), ***trxB***	fig|1351.11559.peg.1149, fig|1351.11559.peg.2376
				metE **-** MULTISPECIES: 5-methyltetrahydropteroyltriglutamate--homocysteine S-methyltransferase [Enterococcus] NCBI Reference Sequence: WP_002362969.1	
	Amino Acids and Derivatives	Lysine, threonine, methionine, and cysteine	Methionine Biosynthesis	Cystathionine beta-synthase (EC 4.2.1.22), ***mccA***	fig|1351.11559.peg.233
	Amino Acids and Derivatives	Lysine, threonine, methionine, and cysteine	Methionine Degradation	Cystathionine beta-synthase (EC 4.2.1.22), ***mccA***	fig|1351.11559.peg.233
	Amino Acids and Derivatives	Alanine, serine, and glycine	Glycine and Serine Utilization	Cystathionine beta-synthase (EC 4.2.1.22), ***mccA***	fig|1351.11559.peg.233
	Cofactors, Vitamins, Prosthetic Groups, Pigments	Folate and pterines	Molybdenum cofactor biosynthesis	Molybdenum ABC transporter, periplasmic molybdenum-binding protein **ModA** (TC 3.A.1.8.1)	fig|1351.11559.peg.1212
	Cofactors, Vitamins, Prosthetic Groups, Pigments	Folate and pterines	Molybdenum cofactor biosynthesis	Molybdenum transport system permease protein **ModB** (TC 3.A.1.8.1)	fig|1351.11559.peg.1213
	Cofactors, Vitamins, Prosthetic Groups, Pigments	Folate and pterines	Molybdenum cofactor biosynthesis	Molybdenum transport ATP-binding protein **ModC** (TC 3.A.1.8.1)	fig|1351.11559.peg.1214
***Staphylococcus carnosus* FDAARGOS_883**					
	Nucleosides and Nucleotides	Pyrimidines	pyrimidine conversions	Thioredoxin reductase (EC 1.8.1.9), ***trxB***	fig|1281.71.peg.283, fig|1281.71.peg.1034, fig|1281.71.peg.2053
	Sulfur Metabolism	Sulfur Metabolism - no subcategory	Thioredoxin-disulfide reductase	Thioredoxin reductase (EC 1.8.1.9), ***trxB***	fig|1281.71.peg.283, fig|1281.71.peg.1034, fig|1281.71.peg.2053
	Amino Acids and Derivatives	Lysine, threonine, methionine, and cysteine	Methionine Biosynthesis	5-methyltetrahydropteroyltriglutamate--homocysteine methyltransferase (EC 2.1.1.14), ***metE***	fig|1281.71.peg.1656
	ofactors, Vitamins, Prosthetic Groups, Pigments	Folate and pterines	5-FCL-like protein	5,10-methylenetetrahydrofolate reductase (EC 1.5.1.20), ***yitJ***	fig|1281.71.peg.1657

	Amino Acids and Derivatives	Lysine, threonine, methionine, and cysteine	Methionine Biosynthesis	5,10-methylenetetrahydrofolate reductase (EC 1.5.1.20), ***yitJ***	fig|1281.71.peg.1657
	Carbohydrates	One-carbon Metabolism	Serine-glyoxylate cycle	5,10-methylenetetrahydrofolate reductase (EC 1.5.1.20), ***yitJ***	fig|1281.71.peg.1657
	Carbohydrates	One-carbon Metabolism	One-carbon metabolism by tetrahydropterines	5,10-methylenetetrahydrofolate reductase (EC 1.5.1.20), ***yitJ***	fig|1281.71.peg.1657
	Amino Acids and Derivatives	Alanine, serine, and glycine	Glycine and Serine Utilization	Cystathionine beta-synthase (EC 4.2.1.22), ***mccA***	fig|1281.71.peg.1730
	Amino Acids and Derivatives	Lysine, threonine, methionine, and cysteine	Methionine Biosynthesis	Cystathionine beta-synthase (EC 4.2.1.22), ***mccA***	fig|1281.71.peg.1730
	Amino Acids and Derivatives	Lysine, threonine, methionine, and cysteine	Methionine Degradation	Cystathionine beta-synthase (EC 4.2.1.22), ***mccA***	fig|1281.71.peg.1730
	-	-	-	**yojG**<br>-<br>bacillithiol biosynthesis deacetylase BshB2 [GenBank: QPT03330.1]	-
	-	-	-	**Catalase**<br>-<br>catalase [Staphylococcus carnosus] GenBank: QPT05115.1<br>catalase [Staphylococcus carnosus] GenBank: QPT02999.1	-
	Cofactors, Vitamins, Prosthetic Groups, Pigments	Folate and pterines	Molybdenum cofactor biosynthesis	Molybdenum ABC transporter, periplasmic molybdenum-binding protein **ModA** (TC 3.A.1.8.1)	fig|1281.71.peg.930
	Cofactors, Vitamins, Prosthetic Groups, Pigments	Folate and pterines	Molybdenum cofactor biosynthesis	Molybdenum transport system permease protein **ModB** (TC 3.A.1.8.1)	fig|1281.71.peg.929
	Cofactors, Vitamins, Prosthetic Groups, Pigments	Folate and pterines	Molybdenum cofactor biosynthesis	Molybdenum transport ATP-binding protein **ModC** (TC 3.A.1.8.1)	fig|1281.71.peg.928<br>fig|1281.71.peg.1071
***Pseudomonas aeruginosa* NBRC 12689**					
	Sulfur Metabolism	Sulfur Metabolism - no subcategory	Thioredoxin-disulfide reductase	Thioredoxin reductase (EC 1.8.1.9), ***trxB***	fig|1123015.134.peg.783<br>fig|1123015.134.peg.2318<br>fig|1123015.134.peg.2411<br>fig|1123015.134.peg.3747<br>fig|1123015.134.peg.4296
	Nucleosides and Nucleotides	Pyrimidines	pyrimidine conversions	Thioredoxin reductase (EC 1.8.1.9), ***trxB***	fig|1123015.134.peg.783<br>fig|1123015.134.peg.2318,<br>fig|1123015.134.peg.2411,<br>fig|1123015.134.peg.3747,<br>fig|1123015.134.peg.4296
	Amino Acids and Derivatives	Lysine, threonine, methionine, and cysteine	Methionine Biosynthesis	5-methyltetrahydropteroyltriglutamate--homocysteine methyltransferase (EC 2.1.1.14), ***metE***	fig|1123015.134.peg.3156
	Amino Acids and Derivatives	Lysine, threonine, methionine, and cysteine	Methionine Biosynthesis	5,10-methylenetetrahydrofolate reductase (EC 1.5.1.20), ***yitJ***	fig|1123015.134.peg.440
	Amino Acids and Derivatives	Lysine, threonine, methionine, and cysteine	Methionine Biosynthesis	Cystathionine beta-synthase (EC 4.2.1.22), ***mccA***	fig|1123015.134.peg.408
	Amino Acids and Derivatives	Lysine, threonine, methionine, and cysteine	Cysteine Biosynthesis	Cystathionine beta-synthase (EC 4.2.1.22), ***mccA***	fig|1123015.134.peg.40
	-	-	-	***yjcL***<br>**-**<br>cystathionine gamma-synthase [GenBank: AKO89911.1]	-
	Stress Response	Oxidative stress	Oxidative stress	Catalase (EC 1.11.1.6)	fig|1123015.134.peg.715<br>fig|1123015.134.peg.2927,<br>fig|1123015.134.peg.3147,<br>fig|1123015.134.peg.4865
	Amino Acids and Derivatives	Lysine, threonine, methionine, and cysteine	Cysteine Biosynthesis	Sulfate transporter, **CysZ**-type	fig|1123015.134.peg.4299
	Amino Acids and Derivatives	Lysine, threonine, methionine, and cysteine	Cysteine Biosynthesis	Sulfate and thiosulfate import ATP-binding protein **CysA** (EC 3.6.3.25)	fig|1123015.134.peg.280
	Amino Acids and Derivatives	Lysine, threonine, methionine, and cysteine	Cysteine Biosynthesis	Sulfate transport system permease protein **CysW**	fig|1123015.134.peg.281

	Amino Acids and Derivatives	Lysine, threonine, methionine, and cysteine	Cysteine Biosynthesis	Sulfate transport system permease protein **CysT**	fig|1123015.134.peg.282
	Amino Acids and Derivatives	Lysine, threonine, methionine, and cysteine	Cysteine Biosynthesis	Sulfate and thiosulfate binding protein **CysP**	fig|1123015.134.peg.283
	Cofactors, Vitamins, Prosthetic Groups, Pigments	Folate and pterines	Molybdenum cofactor biosynthesis	Molybdenum ABC transporter, periplasmic molybdenum-binding protein **ModA** (TC 3.A.1.8.1)	fig|1123015.134.peg.3226
	Cofactors, Vitamins, Prosthetic Groups, Pigments	Folate and pterines	Molybdenum cofactor biosynthesis	Molybdenum transport system permease protein **ModB** (TC 3.A.1.8.1)	fig|1123015.134.peg.3227
	Cofactors, Vitamins, Prosthetic Groups, Pigments	Folate and pterines	Molybdenum cofactor biosynthesis	Molybdenum transport ATP-binding protein **ModC** (TC 3.A.1.8.1)	fig|1123015.134.peg.3228
***Zoogloea ramigera* NBRC 15342**					
	Sulfur Metabolism	Sulfur Metabolism - no subcategory	Thioredoxin-disulfide reductase	Thioredoxin reductase (EC 1.8.1.9), ***trxB***	fig|350.31.peg.136, fig|350.31.peg.690
	Nucleosides and Nucleotides	Pyrimidines	pyrimidine conversions	Thioredoxin reductase (EC 1.8.1.9), ***trxB***	fig|350.31.peg.136, fig|350.31.peg.690
	Carbohydrates	One-carbon Metabolism	One-carbon metabolism by tetrahydropterines	5,10-methylenetetrahydrofolate reductase (EC 1.5.1.20), ***yitJ***	fig|350.31.peg.2355
	Cofactors, Vitamins, Prosthetic Groups, Pigments	Folate and pterines	5-FCL-like protein	5,10-methylenetetrahydrofolate reductase (EC 1.5.1.20), ***yitJ***	fig|350.31.peg.2355
	Stress Response	Oxidative stress	Oxidative stress	Catalase (EC 1.11.1.6)	fig|350.31.peg.184, fig|350.31.peg.3703
	Amino Acids and Derivatives	Lysine, threonine, methionine, and cysteine	Cysteine Biosynthesis	Sulfate and thiosulfate import ATP-binding protein **CysA** (EC 3.6.3.25)	fig|350.31.peg.33
	Amino Acids and Derivatives	Lysine, threonine, methionine, and cysteine	Cysteine Biosynthesis	Sulfate transport system permease protein **CysW**	fig|350.31.peg.32
	Amino Acids and Derivatives	Lysine, threonine, methionine, and cysteine	Cysteine Biosynthesis	Sulfate transport system permease protein **CysT**	fig|350.31.peg.31
	Amino Acids and Derivatives	Lysine, threonine, methionine, and cysteine	Cysteine Biosynthesis	Sulfate and thiosulfate binding protein **CysP**	fig|350.31.peg.30, fig|350.31.peg.56
	Amino Acids and Derivatives	Lysine, threonine, methionine, and cysteine	Cysteine Biosynthesis	Sulfate permease, **SulP**	fig|350.31.peg.185, fig|350.31.peg.440, fig|350.31.peg.1434, fig|350.31.peg.1577
	Cofactors, Vitamins, Prosthetic Groups, Pigments	Folate and pterines	Molybdenum cofactor biosynthesis	Molybdenum ABC transporter, periplasmic molybdenum-binding protein **ModA** (TC 3.A.1.8.1)	fig|350.31.peg.3422
	Cofactors, Vitamins, Prosthetic Groups, Pigments	Folate and pterines	Molybdenum cofactor biosynthesis	Molybdenum transport system permease protein **ModB** (TC 3.A.1.8.1)	fig|350.31.peg.3424
	Cofactors, Vitamins, Prosthetic Groups, Pigments	Folate and pterines	Molybdenum cofactor biosynthesis	Molybdenum transport ATP-binding protein **ModC** (TC 3.A.1.8.1)	fig|350.31.peg.3423
***Klebsiella pneumoniae* HS11286**					
	Sulfur Metabolism	Sulfur Metabolism - no subcategory	Thioredoxin-disulfide reductase	Thioredoxin reductase (EC 1.8.1.9), ***trxB***	fig|1125630.541.peg.1759
	Nucleosides and Nucleotides	Pyrimidines	pyrimidine conversions	Thioredoxin reductase (EC 1.8.1.9), ***trxB***	fig|1125630.541.peg.1759
	Amino Acids and Derivatives	Lysine, threonine, methionine, and cysteine	Methionine Biosynthesis	5-methyltetrahydropteroyltriglutamate--homocysteine methyltransferase (EC 2.1.1.14), ***metE***	fig|1125630.541.peg.167, fig|1125630.541.peg.3079
	Carbohydrates	One-carbon Metabolism	One-carbon metabolism by tetrahydropterines	5,10-methylenetetrahydrofolate reductase (EC 1.5.1.20), ***yitJ***	fig|1125630.541.peg.91
	Carbohydrates	One-carbon Metabolism	Serine-glyoxylate cycle	5,10-methylenetetrahydrofolate reductase (EC 1.5.1.20), ***yitJ***	fig|1125630.541.peg.91
	Amino Acids and Derivatives	Lysine, threonine, methionine, and cysteine	Methionine Biosynthesis	5,10-methylenetetrahydrofolate reductase (EC 1.5.1.20), ***yitJ***	fig|1125630.541.peg.91
	Cofactors, Vitamins, Prosthetic Groups, Pigments	Folate and pterines	5-FCL-like protein	5,10-methylenetetrahydrofolate reductase (EC 1.5.1.20), ***yitJ***	fig|1125630.541.peg.91
	Amino Acids and Derivatives	Lysine, threonine, methionine, and cysteine	S-methylmethionine	Homocysteine S-methyltransferase (EC 2.1.1.10), ***MmuM***	fig|1125630.541.peg.1024

	Amino Acids and Derivatives	Lysine, threonine, methionine, and cysteine	Methionine Biosynthesis	Homocysteine S-methyltransferase (EC 2.1.1.10), ***MmuM***	fig|1125630.541.peg.1024
	Amino Acids and Derivatives	Lysine, threonine, methionine, and cysteine	Methionine Degradation	Cystathionine beta-synthase (EC 4.2.1.22), ***mccA***	fig|1125630.541.peg.4451
	Amino Acids and Derivatives	Lysine, threonine, methionine, and cysteine	Methionine Biosynthesis	Cystathionine beta-synthase (EC 4.2.1.22), ***mccA***	fig|1125630.541.peg.4451
	Amino Acids and Derivatives	Lysine, threonine, methionine, and cysteine	Cysteine Biosynthesis	Cystathionine beta-synthase (EC 4.2.1.22), ***mccA***	fig|1125630.541.peg.445
	Amino Acids and Derivatives	Alanine, serine, and glycine	Glycine and Serine Utilization	Cystathionine beta-synthase (EC 4.2.1.22), ***mccA***	fig|1125630.541.peg.4451
	Sulfur Metabolism	Organic sulfur assimilation	L-Cystine Uptake and Metabolism	Cystathionine gamma-synthase (EC 2.5.1.48), ***yjcL***	fig|1125630.541.peg.85
	Amino Acids and Derivatives	Lysine, threonine, methionine, and cysteine	Methionine Biosynthesis	Cystathionine gamma-synthase (EC 2.5.1.48), ***yjcL***	fig|1125630.541.peg.85
	Stress Response	Oxidative stress	Oxidative stress	Catalase (EC 1.11.1.6)	fig|1125630.541.peg.2088, fig|1125630.541.peg.2714
	Amino Acids and Derivatives	Lysine, threonine, methionine, and cysteine	Cysteine Biosynthesis	Sulfate transporter, **CysZ**-type	fig|1125630.541.peg.3752
	Amino Acids and Derivatives	Lysine, threonine, methionine, and cysteine	Cysteine Biosynthesis	Sulfate and thiosulfate import ATP-binding protein **CysA** (EC 3.6.3.25)	fig|1125630.541.peg.3762
	Amino Acids and Derivatives	Lysine, threonine, methionine, and cysteine	Cysteine Biosynthesis	Sulfate transport system permease protein **CysW**	fig|1125630.541.peg.3763
	Amino Acids and Derivatives	Lysine, threonine, methionine, and cysteine	Cysteine Biosynthesis	Sulfate transport system permease protein **CysT**	fig|1125630.541.peg.3764
	Amino Acids and Derivatives	Lysine, threonine, methionine, and cysteine	Cysteine Biosynthesis	Sulfate and thiosulfate binding protein **CysP**	fig|1125630.541.peg.3765
	Phosphorus Metabolism	Phosphorus Metabolism - no subcategory	Phosphate metabolism	Sodium-dependent phosphate transporter, **PiT**	fig|1125630.541.peg.231
	Cofactors, Vitamins, Prosthetic Groups, Pigments	Folate and pterines	Molybdenum cofactor biosynthesis	Molybdenum ABC transporter, periplasmic molybdenum-binding protein **ModA** (TC 3.A.1.8.1)	fig|1125630.541.peg.1567
	Cofactors, Vitamins, Prosthetic Groups, Pigments	Folate and pterines	Molybdenum cofactor biosynthesis	Molybdenum transport system permease protein **ModB** (TC 3.A.1.8.1)	fig|1125630.541.peg.1568
	Cofactors, Vitamins, Prosthetic Groups, Pigments	Folate and pterines	Molybdenum cofactor biosynthesis	Molybdenum transport ATP-binding protein **ModC** (TC 3.A.1.8.1)	fig|1125630.541.peg.1569
***Streptococcus thermophilus* ATCC 19258**					
	Sulfur Metabolism	Sulfur Metabolism - no subcategory	Thioredoxin-disulfide reductase	Thioredoxin reductase (EC 1.8.1.9), ***trxB***	fig|1308.2696.peg.2039, fig|1308.2696.peg.2286
	Nucleosides and Nucleotides	Pyrimidines	pyrimidine conversions	Thioredoxin reductase (EC 1.8.1.9), ***trxB***	fig|1308.2696.peg.2039, fig|1308.2696.peg.2286
	Amino Acids and Derivatives	Lysine, threonine, methionine, and cysteine	Methionine Biosynthesis	5-methyltetrahydropteroyltriglutamate--homocysteine methyltransferase (EC 2.1.1.14), ***metE***	fig|1308.2696.peg.361, fig|1308.2696.peg.362, fig|1308.2696.peg.363, fig|1308.2696.peg.1384, fig|1308.2696.peg.1534, fig|1308.2696.peg.1535
	Amino Acids and Derivatives	Lysine, threonine, methionine, and cysteine	Methionine Biosynthesis	5,10-methylenetetrahydrofolate reductase (EC 1.5.1.20), ***yitJ***	fig|1308.2696.peg.1385
	Amino Acids and Derivatives	Lysine, threonine, methionine, and cysteine	Methionine Biosynthesis	Homocysteine S-methyltransferase (EC 2.1.1.10), ***MmuM***	fig|1308.2696.peg.1125
	Amino Acids and Derivatives	Lysine, threonine, methionine, and cysteine	S-methylmethionine	Homocysteine S-methyltransferase (EC 2.1.1.10), ***MmuM***	fig|1308.2696.peg.1125
	Amino Acids and Derivatives	Lysine, threonine, methionine, and cysteine	Methionine Biosynthesis	Cystathionine gamma-synthase (EC 2.5.1.48), ***yjcL***	fig|1308.2696.peg.817
***Stenotrophomonas maltophilia* NCTC 10257**					

	Nucleosides and Nucleotides	Pyrimidines	pyrimidine conversions	Thioredoxin reductase (EC 1.8.1.9), ***trxB***	fig|40324.3640.peg.2285, fig|40324.3640.peg.4403
	Sulfur Metabolism	Sulfur Metabolism - no subcategory	Thioredoxin-disulfide reductase	Thioredoxin reductase (EC 1.8.1.9), ***trxB***	fig|40324.3640.peg.2285, fig|40324.3640.peg.4403
	Amino Acids and Derivatives	Lysine, threonine, methionine, and cysteine	Methionine Biosynthesis	5-methyltetrahydropteroyltriglutamate--homocysteine methyltransferase (EC 2.1.1.14), ***metE***	fig|40324.3640.peg.2436
	Amino Acids and Derivatives	Lysine, threonine, methionine, and cysteine	Methionine Biosynthesis	5,10-methylenetetrahydrofolate reductase (EC 1.5.1.20), ***yitJ***	fig|40324.3640.peg.709, fig|40324.3640.peg.2432
	Amino Acids and Derivatives	Lysine, threonine, methionine, and cysteine	Methionine Degradation	Cystathionine beta-synthase (EC 4.2.1.22), ***mccA***	fig|40324.3640.peg.560
	Amino Acids and Derivatives	Lysine, threonine, methionine, and cysteine	Methionine Biosynthesis	Cystathionine beta-synthase (EC 4.2.1.22), ***mccA***	fig|40324.3640.peg.560
	Amino Acids and Derivatives	Lysine, threonine, methionine, and cysteine	Methionine Biosynthesis	Cystathionine gamma-synthase (EC 2.5.1.48), ***yjcL***	fig|40324.3640.peg.3492
	Stress Response	Oxidative stress	Oxidative stress	Catalase (EC 1.11.1.6)	fig|40324.3640.peg.324, fig|40324.3640.peg.1371, fig|40324.3640.peg.3442

Table S4. BLAST similarity search analyses of identified genes related to selenium uptake and metabolism in strain BLN313.

Gene Name	Nearest homologs	Query coverage (%)	Identity (%)
trxB	*Bacillus licheniformis* FDAARGOS (CP065647.1)	100	100
	Bacillus sp. FSL P2-0026 (CP151959.1)	100	100
	Bacillus sp. H15-1 (CP018249.1)	100	100
	Bacillus sp. 1s-1 (CP022874.1)	100	100
	Bacillus sp. PAMC28748 (CP060192.1)	100	99.91
	Bacillus paralicheniformis NG-ABK-31 (CP104093.1)	100	96.32
metE	*Bacillus licheniformis* FDAARGOS, *metE* gene (CP065647.1)	100	100
	Bacillus sp. FSL P2-0026, *metE* gene (P151959.1)	100	100
	Bacillus sp. FSL K6-1109, *metE* gene (CP150273.1)	100	100
	Bacillus sp. PAMC28748, *metE* gene (CP060192.1)	100	100
	Bacillus sp. H15-1, *metE* gene (CP018249.1)	100	99.96
	Bacillus sp. 1s-1, *metE* gene (CP022874.1)	100	99.96
	Bacillus paralicheniformis ZAP17, *metE* gene (CP049698.1)	100	94.89
yitJ	*Bacillus licheniformis* FDAARGOS, *yitJ* gene (CP065647.1)	100	100
	Bacillus sp. FSL P2-0026, *yitJ* gene (P151959.1)	100	100
	Bacillus sp. FSL K6-1109, *yitJ* gene (CP150273.1)	100	100
	Bacillus sp. H15-1, *yitJ* gene (CP018249.1)	100	100
	Bacillus sp. 1s-1, *yitJ* gene (CP022874.1)	100	100
	Bacillus sp. PAMC28748, *yitJ* gene (CP060192.1)	100	99.95
	Bacillus paralicheniformis ZAP17, *yitJ* gene (CP049698.1)	100	96.17
MmuM	*Bacillus licheniformis* FDAARGOS, *mmuM gene* (CP065647.1)	100	100
	Bacillus sp. FSL P2-0026, *mmuM gene* (P151959.1)	100	100
	Bacillus sp. FSL K6-1109, *mmuM gene* (CP150273.1)	100	100
	Bacillus sp. H15-1, *mmuM gene* (CP018249.1)	100	100
	Bacillus sp. 1s-1, *mmuM gene* (CP022874.1)	100	100
	Bacillus sp. PAMC28748, *mmuM gene* (CP060192.1)	100	100
	Bacillus paralicheniformis ZAP17, *mmuM gene* (CP049698.1)	100	93.13
mccA	*Bacillus licheniformis* FDAARGOS, cysteine synthase family protein (CP065647.1)	100	100
	Bacillus sp. FSL P2-0026, cysteine synthase family protein (P151959.1)	100	100
	Bacillus sp. FSL K6-1109, cysteine synthase family protein (CP150273.1)	100	100
	Bacillus sp. H15-1, cysteine synthase (CP018249.1)	100	100
	Bacillus licheniformis WX-02, cysteine synthase YrhA (CP012110.1)	100	100
	Bacillus sp. 1s-1, cysteine synthase (CP022874.1)	100	100
	Bacillus licheniformis ATCC 14580, cysteine synthase family protein (CP000002.3)	100	100
	Bacillus sp. FSL M8-0359, cysteine synthase (CP150246.1)	100	99.89
	Bacillus sp. PAMC28748, cysteine synthase family protein (CP060192.1)	100	99.89
	Bacillus paralicheniformis ZAP17, cysteine synthase family protein (CP049698.1)	100	94.37
yjcL	*Bacillus licheniformis* FDAARGOS, *cystathionine beta-lyase* (CP065647.1)	100	100
	Bacillus sp. FSL P2-0026, *cystathionine beta-lyase*, (P151959.1)	100	100
	Bacillus sp. H15-1, *yjcL gene* (CP018249.1)	100	100
	Bacillus sp. 1s-1, *yjcL gene* (CP022874.1)	100	100
	Bacillus sp. FSL K6-1109, *cystathionine beta-lyase* (CP150273.1)	100	100
	Bacillus sp. FSL M8-0133, *cystathionine beta-lyase* (CP150249.1)	100	100
	Bacillus sp. PAMC28748, *cystathionine beta-lyase* (CP060192.1)	100	100
	Bacillus paralicheniformis CamBx3, *cystathionine beta-lyase* (CP110812.1)	100	96.62
ypjG	*Providencia huaxiensis* WCHPr000369 (CP031123.2), PIG-L deacetylase family protein	100	100
	Providencia huaxiensis PR66 (CP178221.1), PIG-L deacetylase family protein	100	94.35
yojG	*Bacillus licheniformis* 14ADL4, putative N-acetyl-alpha-D-glucosaminyl L-malate deacetylase (CP026673.1)	100	100
	Bacillus sp. FSL P2-0026, bacillithiol biosynthesis deacetylase BshB2 (CP151959.1)	100	100
	Bacillus licheniformis MGMM9, bacillithiol biosynthesis deacetylase BshB2 (CP130465.1)	100	100
**SulP-I**	*Bacillus licheniformis* 14ADL4-Sulfate permease CysP (CP026673.1)	100	100
	Bacillus sp. 1s-1-inorganic phosphate transporter (CP022874.1)	100	100
	Bacillus sp. FSL M8-0133- inorganic phosphate transporter (CP150249.1)	100	100
	Bacillus sp. PAMC28748-inorganic phosphate transporter (CP060192.1)	100	100
**SulP-II**	*Bacillus licheniformis* 14ADL4-PiT (CP026673.1)	100	100
	Bacillus sp. FSL P2-0026-inorganic phosphate transporter (CP151959.1)	100	100
	Bacillus licheniformis MGMM9- anion permease (CP130465.1)	100	100
**Sulfate transporter/permease *ybaR***	Bacillus sp. FSL P2-0026-SulP family inorganic anion transporter (CP151959.1)	100	100
	Bacillus licheniformis MGMM9-SulP family inorganic anion transporter (CP130465.1)	100	100
	Bacillus licheniformis FDAARGOS 923- SulP family inorganic anion transporter (CP065647.1)	100	100
**Sulfate transporter/permease *yvdB***	*Bacillus* sp. FSL P2-0026-solute carrier family 23 protein (CP151959.1)	100	100
	Bacillus licheniformis FDAARGOS 923- STAS domain-containing protein (CP065647.1)	100	100
	Bacillus licheniformis MPB04-SulP family inorganic anion transporter (CP177039.1)	100	100
	Bacillus sp. FSL K6-1109-solute carrier family 23 protein (CP150273.1)	100	100
	Bacillus sp. PAMC28748-STAS domain-containing protein (CP060192.1)	100	100
**PiT-isomerI**	*Bacillus licheniformis* ATCC 14580, Na/Pi cotransporter family protein (CP140161.1)	100	100
	Bacillus licheniformis T5, Na/Pi cotransporter family protein (CP124852.1)	100	100
	Bacillus sp. 1s-1, sodium-dependent phosphate transporter (CP022874.1)	100	100
	Bacillus licheniformis FDAARGOS_923, Na/Pi cotransporter family protein (CP065647.1)	100	100
**PiT-isomerII**	*Bacillus licheniformis* FUA2151, Na/Pi symporter (CP143963.1)	100	100
	Bacillus licheniformis CP6, Na/Pi cotransporter family protein (CP049330.1)	100	99.79
	Bacillus sp. FSL K6-8391, Na/Pi symporter (CP151999.1)	100	99.79
	Bacillus sp. PAMC28748, Na/Pi symporter (CP060192.1)	100	99.68
