# Supplementary material for: Integrated Optimization, Genomic Characterization, and Functional Evaluation of Biogenic Selenium Nanoparticles from Bacillus licheniformis BLN313: Antibacterial and Anticancer Potential
Source: Int J Mol Sci. 2026 Aug 31;27(17):7792. doi: 10.3390/ijms27177792 (PMC13566226; doi:10.3390/ijms27177792)
Supplement: Supplementary file 1 [file ijms-27-07792-s001.zip › Supplementary tables 1 and 2.pdf]

Supplementary Table S1. Complete list of Reagents and their formula/grade and suppliers.

**Reagent / Material**	**Formula / Description**	**Purity / Grade**	**Supplier / Company**
Sodium selenite	Na_2SeO_3	≥98.0%, analytical grade	Sinopharm Chemical Reagent Co., Ltd, China
Sodium hydroxide	NaOH	≥96.0%, analytical grade	Sinopharm Chemical Reagent Co., Ltd, China
Sodium chloride	NaCl	≥99.5%, analytical grade	Sinopharm Chemical Reagent Co., Ltd, China
Hydrochloric acid	HCl	≥37%, concentrated	Sinopharm Chemical Reagent Co., Ltd, China
Hydrogen peroxide	H_2O_2	≥35.0%	Sinopharm Chemical Reagent Co., Ltd, China
Tryptone	Complex protein digest	Biological grade	Beijing Aoboxing Biotechnology Co., Ltd, China
Agar powder	Polysaccharide agar	Biological grade	Beijing Kangbeisi Technology Co., Ltd, China
Yeast extract LP0021	Complex biological extract	Biological grade	Oxoid, France
Glycerol	$C_3H_8O_3$	Analytical grade	Sinopharm / Sigma / Solarbio, China
HNO_3	HNO_3	Analytical grade	Sinopharm / Sigma / Solarbio, China

Reagent / Material	Formula / Description	Purity / Grade	Supplier / Company
Lysozyme	Enzyme/protein	Analytical grade	Solarbio / Sigma, China
Tris-HCl	$C_4H_{11}NO_3 \cdot HCl$	Molecular biology grade	Solarbio / Sigma, China
SDS	$C_{12}H_{25}NaO_4S$	Analytical grade	Solarbio / Sigma, China
1-Octanol	$C_8H_{18}O$	Analytical grade	Sinopharm / Sigma, China
Chloroform	$CHCl_3$	Analytical grade	Sinopharm / Sigma, China
Absolute ethanol	C_2H_6O	Analytical grade	Sinopharm / Sigma, China
KBr	KBr	FTIR grade	Sinopharm / Sigma, China
Ciprofloxacin	$C_{17}H_{18}FN_3O_3$	Analytical grade	Solarbio / Sigma, China
DMEM	Commercial cell culture medium	Cell culture grade	Gibco / Thermo Fisher Scientific
FBS	Biological supplement	Cell culture supplement	Gibco / Thermo Fisher Scientific
Penicillin–streptomycin	Commercial antibiotic mixture	Cell culture grade	Gibco / Thermo Fisher Scientific / Life Technologies
L-glutamine	$C_5H_{10}N_2O_3$	Cell culture grade	Gibco / Thermo Fisher Scientific
NEAA	Commercial formulation	Cell culture supplement	Gibco / Thermo Fisher Scientific
Trypsin–EDTA	Commercial enzyme solution	0.25% solution	Gibco / Thermo Fisher Scientific
MTT	$C_{18}H_{16}BrN_5S$	12 mM solution / cell culture grade	Life Technologies / Thermo Fisher Scientific

Reagent / Material	Formula / Description	Purity / Grade	Supplier / Company
DMSO	C_2H_6OS	Analytical / cell culture grade	Sigma / Solarbio / Thermo Fisher

Supplementary Table S2. DLS and zeta potential measurements of BLN313-SeNPs

Replicate	Zeta potential (mV)	Zeta deviation (mV)	Conductivity (mS/cm)
1	−21.0	5.13	0.601
2	−20.7	5.31	0.611
3	−20.7	4.15	0.618
**Mean ± SD**	**−20.8 ± 0.2**	**4.86 ± 0.62**	**0.610 ± 0.009**

Measurement	Z-average diameter (nm)	PDI	D(10) (nm)	D(50) (nm)	D(90) (nm)
Replicate 1	166.0	0.028	124	170	234
Replicate 2	166.0	0.006	125	169	230
Replicate 3	165.3	0.080	127	164	214
**Mean ± SD**	**165.8 ± 0.4**	**0.038± 0.038**	**125.3 ± 1.5**	**167.7 ± 3.2**	**226.0 ± 10.6**
